# Endogenous Sulfane Sulfur Mediates the Oxidative Stress Response Process in *Pseudomonas aeruginosa*

**DOI:** 10.3390/antiox15060696

**Published:** 2026-05-31

**Authors:** Weining Sun, Xiaoqian Guo, Feng Chen, Guangyu Liu

**Affiliations:** Zhejiang Key Laboratory of Medical Epigenetics, Department of Immunology and Pathogen Biology, School of Basic Medical Sciences, Hangzhou Normal University, Hangzhou 311121, Chinaguoxq0216@163.com (X.G.); zoezilla@163.com (F.C.)

**Keywords:** oxidative stress, *P. aeruginosa*, OxyR, sulfane sulfur, persulfidation

## Abstract

Sulfane sulfur species are increasingly recognized as integral cellular components involved in signaling pathways and cytoprotection against oxidative stress in mammals. While their production in bacteria has been extensively studied, their functional role in bacterial oxidative stress defense remains poorly understood. Here, we demonstrate that sulfane sulfur generated by sulfide: quinone oxidoreductase decreases H_2_O_2_ sensitivity in *Pseudomonas aeruginosa* PAO1. Notably, this protective mechanism does not depend on sulfane sulfur acting as a direct H_2_O_2_ scavenger via nucleophilic reactions. Through persulfidation proteomic profiling, we reveal that persulfidation is a prominent post-translational modification in *P. aeruginosa*, reflecting the prevalence of deprotonated sulfane sulfur species. These species modify cysteine residues in proteins, including the well-known oxidative stress regulator OxyR. Specifically, sulfane sulfur modifies OxyR at Cys199 to form persulfidated OxyR C199-SSH, contributing to a single-Cys activated state that modulates promoter activity and DNA-binding affinity. Furthermore, sulfane sulfur-mediated persulfidation protects the critical cysteine residue of LpdG, a ROS-vulnerable dihydrolipoamide dehydrogenase, from irreversible overoxidation. Although LpdG is not part of the canonical H_2_O_2_-scavenging system, its preservation is essential for cell viability under oxidative stress. These findings establish endogenous sulfane sulfur species as key mediators of antioxidant defense in *P. aeruginosa*.

## 1. Introduction

Oxygen (O_2_), while serving as the most efficient terminal electron acceptor in aerobic respiration, inevitably generates reactive oxygen species (ROS) through electron leakage from the respiratory chain [1]. These ROS, including superoxide anion (O_2_•^−^), hydroxyl radical (OH•), and hydrogen peroxide (H_2_O_2_), not only arise endogenously but also accumulate extrinsically through exposure to antibiotics and host immune systems [2]. Together, these dual sources of ROS impose a persistent oxidative challenge on bacterial cells, causing irreversible damage to DNA and thiol-containing proteins [3,4,5].

In Gram-negative bacteria, the LysR family transcription factor OxyR plays a central role in orchestrating oxidative stress responses. When intracellular H_2_O_2_ levels exceed a threshold, two conserved cysteine residues (Cys199/Cys208) in the regulatory domain of OxyR undergo disulfide bond formation, triggering conformational changes that facilitate promoter binding and regulon activation [6,7,8,9].

Beyond canonical ROS response pathways, H_2_S derived from conserved bacterial cysteine degradation pathways has been implicated in oxidative stress resistance in *Escherichia coli* [10,11]. However, H_2_S exhibits limited capacity for direct thiol reactivity due to its chemical nature, casting doubt on its role in direct H_2_O_2_ detoxification. Sulfane sulfur encompasses diverse chemical forms, such as RSSH, RSS_n_H (*n* ≥ 1), HSS_n_H, and RSS_n_R (*n* ≥ 1), with deprotonated sulfane sulfur predominating in the intracellular milieu. Compared with H_2_S, deprotonated sulfane sulfur is more electrophilic, thereby facilitating persulfidation of reactive cysteine residues [12,13,14,15]. In mammalian cells, sulfane sulfur has emerged as an important mediator of ROS stress responses [16,17,18].

While ROS directly oxidize protein thiols through a hierarchical cascade, including reversible sulfenic acid (-SOH), as well as irreversible oxidation to sulfinic acid (-SO_2_H) and sulfonic acid (-SO_3_H) (Figure S1) [4], which ultimately leads to irreversible protein inactivation, sulfane sulfur-mediated persulfidation circumvents this irreversible fate [16]. By covalently modifying Cys residues, persulfides stabilize the thiol redox state, thereby preserving enzyme activity and cellular homeostasis (Figure S1) [19,20].

Bacterial reactive sulfur species (RSS) biosynthesis operates through two major pathways. The first pathway is linked to cysteine metabolism and proceeds via the mercaptopyruvate route: L-cysteine is first converted to 3-mercaptopyruvate by L-cysteine aminotransferase (CAT); subsequently, 3-mercaptopyruvate sulfurtransferase (MST) transfers a sulfur atom to a thiol acceptor such as glutathione, generating glutathione persulfide (GSSH) as a key sulfane sulfur species [21]. In some bacteria, methionine can also contribute to this pathway after being converted to cysteine via the transsulfuration pathway, which involves cystathionine β-synthase (CBS) and cystathionine γ-lyase (CSE) [22]. The second pathway is mediated by sulfide:quinone oxidoreductase (SQR). This quinone-dependent route, exemplified in *Pseudomonas* spp., directly oxidizes H_2_S to generate active sulfane sulfur via two-electron transfer to ubiquinone [23]. Despite these mechanistic insights, the physiological functions of sulfane sulfur in bacterial oxidative stress adaptation remain incompletely understood.

Survival of pathogenic bacteria in aerobic niches and host tissues hinges critically on effective ROS stress defenses. *P. aeruginosa*, a paradigmatic opportunistic pathogen, exemplifies this challenge by chronically infecting immunocompromised patients and causing life-threatening conditions such as cystic fibrosis and respiratory failure, both of which contribute substantially to intensive care unit (ICU) morbidity [24,25]. Notably, *P. aeruginosa* harbors diverse sulfane sulfur biosynthesis pathways, enabling dynamic redox regulation. Sulfane sulfur has been implicated in modifying conserved Cys residues of MexR, the master regulator of the efflux pump MexAB-OprM, and the quorum-sensing transcriptional regulator LasR, demonstrating its pivotal role in modulating transcriptional repression in *P. aeruginosa* [26,27]. While *Escherichia coli* OxyR senses sulfane sulfur using Cys199 as a persulfidation substrate [28], the functional relevance of this modification in oxidative stress remains unexplored. *P. aeruginosa* PAO1 therefore represents an ideal model for dissecting the physiological functions of sulfane sulfur in bacteria during oxidative stress responses.

This study establishes sulfane sulfur as a dual-function regulator of oxidative stress in *P. aeruginosa* PAO1. First, sulfane sulfur orchestrates hierarchical transcriptional activation of the OxyR regulon through persulfidation of its conserved Cys199 residue, thereby conferring enhanced tolerance to H_2_O_2_. Second, sulfane sulfur safeguards redox-sensitive proteins by selectively persulfidating their cysteine residues, thereby mitigating irreversible oxidative damage. Collectively, these findings reveal a previously unappreciated sulfane sulfur-dependent antioxidant paradigm in pathogenic bacteria.

## 2. Materials and Methods

### 2.1. Bacterial Strains, Plasmids and Culture Conditions

The bacterial strains and plasmids used in this study are listed in Table 1, and the primers are listed in Table S1. For genetic manipulation, *P. aeruginosa* and *E. coli* strains were cultivated under aerobic conditions in Lennox broth (LB; Sangon Biotech, Shanghai, China), which contained tryptone (10 g/L), yeast extract (5 g/L), and NaCl (5 g/L), and incubated at 37 °C. When required, growth media were supplemented with 2,6-diaminopimelic acid (DAP) at a final concentration of 0.3 mM and gentamicin at a final concentration of 60 μg/mL.

*P. aeruginosa* strain growth was monitored by measuring optical density at 600 nm (OD_600_) in triplicate. For aerobic cultivation, fresh medium was inoculated with overnight cultures to an initial OD_600_ of 0.01 and incubated at 37 °C with shaking at 250 rpm.

### 2.2. In-Frame Deletion Mutagenesis and Inducible Gene Expression

In-frame deletion mutants were constructed using *att*-based fusion PCR in combination with SacB-based counter-selection as previously described [7]. Briefly, two DNA fragments flanking the target gene were amplified using primers containing *attB* sites and subsequently fused by a second round of PCR using the outer primers from the first amplification. The resulting fusion fragments were introduced into plasmid pHGM01 using the Gateway BP Clonase II enzyme mix (Invitrogen, Carlsbad, CA, USA) according to the manufacturer’s instructions. Mutagenesis vectors were maintained in the *E. coli* DAP auxotroph strain WM3064 and subsequently transferred into the relevant *P. aeruginosa* strains via conjugation. Integration of deletion constructs into the chromosome was selected on gentamicin-containing medium and confirmed by PCR. Verified transconjugants were grown in LB without NaCl and subsequently plated on LB containing 10% sucrose. Gentamicin-sensitive and sucrose-resistant colonies were screened by PCR for the target deletions and further verified by sequencing the mutated regions.

All mutants used in this study, including those constructed previously, were verified through genetic complementation. Newly constructed mutants were complemented using plasmid pHGEN-Ptac, which carries an isopropyl β-D-1-thiogalactoside (IPTG)-inducible Ptac promoter. Target genes were amplified by PCR and cloned into pHGEN-Ptac, and following sequence verification, the resulting vectors were transferred into the relevant strains via conjugation. These pHGEN-Ptac vectors were also employed for inducible gene expression.

### 2.3. Analytical Techniques for Determination of Sulfur Compounds

Intracellular sulfane sulfur levels in bacteria were determined using the SSP4 (sulfane sulfur probe 4) method https://www.dojindo.com/manual/SB10/ (accessed on 27 May 2026) [28]. Bacterial cells at the mid-exponential phase (OD_600_ of approximately 0.5) were harvested, washed, and resuspended. The bacterial suspension was then mixed with SSP4 (final concentration, 10 μM) and cetyltrimethylammonium bromide (CTAB, 0.5 mM) and incubated in the dark at 30 °C for 20 min. Following centrifugation to remove the supernatant, cells were washed twice and resuspended in phosphate-buffered saline (PBS). Fluorescence intensity generated by the reaction between SSP4 and sulfane sulfur was measured using a Synergy 2 Pro200 Multi-Detection Microplate Reader (Tecan, Männedorf, Switzerland) at excitation and emission wavelengths of 482 nm and 515 nm, respectively. A negative control consisting of buffer and the SSP4 probe was used to subtract the background signal. A standard curve was generated using sodium tetrasulfide (Na_2_S_4_) as the standard, following the same incubation and fluorescence measurement conditions. The resulting linear equation (R^2^ ≥ 0.99) was used to calculate sulfane sulfur concentrations.

H_2_S production in *P. aeruginosa* strains was monitored using a lead acetate detection method [29]. Paper strips saturated with 2% lead acetate (Pb(Ac)_2_) were affixed to the inner wall of culture tubes above the liquid culture level. Fresh LB medium was inoculated with overnight cultures to an OD_600_ of 0.01 and incubated with shaking at 37 °C for approximately 24 h. Paper strips were carefully removed from the tubes and photographed at the end of the incubation period.

### 2.4. Promoter Activity Assay

Promoter activity was determined using a single-copy integrative *lac*Z reporter system as described previously [7,8], with minor modifications.

Briefly, DNA fragments encompassing approximately 300 bp upstream of the target gene start codon were amplified by PCR, cloned into the reporter vector pHGEI01, and verified by DNA sequencing. The resulting recombinant plasmid was transferred from *E. coli* WM3064 into the relevant strains by conjugation, where it integrated into the chromosome as a single copy. Cells were harvested during mid-exponential phase, washed once with PBS, and disrupted by sonication. After centrifugation at 4 °C to remove cell debris, the soluble protein fraction was mixed with an equal volume of o-nitrophenyl-β-D-galactopyranoside (ONPG, 4 mg/mL) and incubated at 37 °C. β-Galactosidase activity was monitored by measuring the absorbance at 420 nm using a Synergy 2 Pro200 Multi-Detection Microplate Reader (Tecan, Männedorf, Switzerland).

Enzyme activity was calculated as Miller units using the following formula:

$$\mathrm{Miller}\;\mathrm{units}=\frac{{\mathrm{OD}}_{420}\times 1000}{V\times {\mathrm{OD}}_{600}\times t}$$

where:

${\mathrm{OD}}_{420}$ = absorbance of the reaction product,

V = reaction volume (mL),

${\mathrm{OD}}_{600}$ = cell density before lysis,

t = reaction time (min).

### 2.5. Assay for H_2_O_2_ Consumption

H_2_O_2_ concentration was determined using the FOX assay [7]. FOX I solution contained 100 mM sorbitol and 125 μM xylenol orange, and FOX II solution contained 25 mM ferrous ammonium sulfate and 2.5 M sulfuric acid. Fresh FOX reagent was prepared by mixing FOX I and FOX II at a ratio of 100:1 (*v*/*v*) immediately before use. *P. aeruginosa* cells harvested at mid-exponential phase were washed twice with PBS (pH 7.0) and resuspended to an OD_600_ of 0.1. H_2_O_2_ was added to a final concentration of 0.1 mM, and the suspension was incubated at 37 °C. Aliquots were withdrawn at 0, 5, 15, and 20 min after H_2_O_2_ addition, and cells were immediately removed by filtration (0.22-μm filter). For each time point, 20 μL of filtrate was mixed with 180 μL of FOX reagent in a 96-well plate and incubated at 37 °C for 30 min. Absorbance was measured at 562 nm using a Synergy 2 Pro200 Multi-Detection Microplate Reader (Tecan, Männedorf, Switzerland). H_2_O_2_ concentration was calculated from a standard curve.

### 2.6. Immunoblotting Assays

Purified protein concentration was determined using the bicinchoninic acid assay. Samples were loaded onto SDS-polyacrylamide gels, stained with Coomassie brilliant blue, or electrophoretically transferred to polyvinylidene difluoride (PVDF) membranes according to the manufacturer’s instructions (Bio-Rad, Shanghai, China). Protein transfer was conducted at 100 V for 1 h using a Criterion blotter (Bio-Rad, Shanghai, China). Membranes were probed with rabbit polyclonal antibodies, followed by incubation with goat anti-rabbit IgG conjugated to horseradish peroxidase (HRP) at a 1:10,000 dilution as the secondary antibody. Signals were detected using a chemiluminescence Western blotting kit (Beyotime, Shanghai, China) and visualized with the UVP imaging system. Rabbit polyclonal antibody against a fragment of *P. aeruginosa* OxyR was provided by the manufacturer (GenScript, Nanjing, China) and used for immunoblotting analyses.

### 2.7. Growth and Susceptibility to H_2_O_2_

Disk diffusion assays were performed on *P. aeruginosa* strains to assess their susceptibility to oxidative stress. Bacterial cultures at mid-exponential phase (200 µL) were mixed with LB medium and poured onto plates. After the plates solidified, sterile filter paper disks were placed on the surface, and 10 μL of 1 M H_2_O_2_ was applied dropwise to each disk. Following overnight incubation at 37 °C, the diameter of the inhibition zone was measured.

### 2.8. Transposon Mutagenesis and Screening

To construct a random transposon insertion library in the Δ*sqr12* mutant strain, conjugation was first performed between *E. coli* WM3064 carrying the transposon vector pFAC, which harbors a strong promoter within the transposable region, and the recipient Δ*sqr12* strain [30]. Following conjugation, the cells were diluted to an OD_600_ of 0.3 and exposed to varying concentrations of H_2_O_2_ (0.5 mM, 1 mM, and 2 mM) for 20 min, then plated on gentamicin-containing medium to select for transposon insertions. From the resulting random mutant library, colonies with substantially larger sizes than the average were selected as putative suppressor mutants of Δ*sqr12*. Finally, the transposon insertion sites in these mutants were determined by arbitrary PCR.

### 2.9. Expression, Purification, and Persulfidation of P. aeruginosa OxyR and LpdG Proteins

OxyR and LpdG proteins were expressed and purified as His_6_-tagged proteins. Briefly, plasmid pET28a carrying the *oxyR* or *lpdG* gene with His_6_-tag-encoding sequences added to the 3′ end was introduced into *E. coli* BL21. *E. coli* BL21 cells transformed with pET28a derivatives harboring the target genes were cultivated in LB to the mid-logarithmic phase at 37 °C and then induced with 0.3 mM IPTG for 10 h at 18 °C to achieve high-level protein expression. Cells were harvested by centrifugation at 5000 rpm for 15 min and resuspended in binding buffer (10 mM Tris-HCl, 150 mM NaCl, pH 8.0). Cell pellets were lysed using a French press, and target proteins were purified from crude cell lysates using nickel-ion affinity chromatography (GE Healthcare, Maidstone, UK). After contaminant proteins were removed by washing with Wash Buffer A containing 20 mM imidazole, the His-tagged OxyR or LpdG proteins were eluted using a linear gradient of Elution Buffer B containing 300 mM imidazole. Elution fractions were collected and analyzed by SDS-PAGE.

To prepare the sulfane sulfur solution, NaHS, elemental sulfur, and NaOH (44.8 mg, 25.6 mg, and 32 mg, respectively) were added to 20 mL of deoxygenated ultrapure water in an anaerobic bottle, sealed immediately, and incubated at 37 °C until the sulfur powder was completely dissolved. The SSP4 probe was applied to quantify the concentration of the sulfane sulfur solution, and a standard curve was generated using sodium tetrasulfide (Na_2_S_4_) as the reference standard.

Purified target protein (10 µM) was pre-reduced with 1 mM dithiothreitol (DTT) for 30 min. A desalting column was used to remove unreacted components, and the target protein was then treated with sulfane sulfur at the indicated concentrations for 30 min at room temperature to generate persulfidated protein.

### 2.10. Site-Directed Mutagenesis

Plasmid pHGE-Ptac-*lpdG* was used as the template for site-directed mutagenesis with a QuikChange II XL site-directed mutagenesis kit (Agilent, Santa Clara, CA, USA) to generate LpdG proteins carrying point mutations. Mutated PCR products were generated with primers, subsequently digested by DpnI, and transformed into *E. coli* WM3064. After sequencing verification, the resulting plasmid was transferred into the *P. aeruginosa* strains by conjugation.

Chromosomal OxyR and LpdG point variants were constructed as follows: the mutant fragments were cloned into integrative vector pHGM01 and verified by sequencing. The resulting constructs were integrated into the chromosome of the Δ*oxyR or* Δ*lpdG* strain via homologous recombination at the *oxyR* or *lpdG* locus, placing the mutant alleles under the control of the native *oxyR* promoter. Integrants were confirmed by PCR and sequencing.

### 2.11. EMSA of OxyR Protein and the DNA Probe of katA Promoter Regions

EMSA was performed to detect the interactions of OxyR protein and the DNA probe of *katA* promoter regions. Briefly, a biotin-labeled DNA probe containing the *katA* promoter binding site was prepared by heating to 95 °C followed by slow annealing to form double-stranded probes. For the binding reaction, the purified OxyR protein at various concentrations was incubated with 20 fmol of biotin-labeled probe, 1 µg poly(dI-dC), and binding buffer (4 mM Tris-HCl pH 8.0, 40 mM NaCl, 4 mM MgCl_2_, and 4% glycerol) in a total volume of 20 µL. The reaction mixture was incubated at room temperature for 20 min to allow protein-probe binding. After incubation, the DNA-protein complexes were loaded onto a 6% native polyacrylamide gel that had been pre-run in 0.5× TBE buffer at 4 °C and 80 V for 30 min. Electrophoresis was carried out at 4 °C under constant voltage of 100 V for approximately 90 min. Following electrophoresis, protein-DNA complexes were transferred to a nylon membrane (GE Healthcare, Maidstone, UK) and crosslinked by UV irradiation at 120 mJ/cm^2^. Finally, the biotin-labeled probes were detected using a chemiluminescent method [8].

### 2.12. Thiol-Labeled Peptide Enrichment

Protein samples were precipitated using the acetone precipitation method. Following redissolution, samples were labeled with 200 mM maleimide-biotin, subjected to overnight acetone precipitation at low temperature, and digested with trypsin overnight before affinity purification to eliminate false positives. Equal amounts of peptide fragments were mixed with 100 µL of high-capacity NeutrAvidin beads at room temperature for 1 h. Beads were washed five times with 1 mL of wash buffer (0.2% SDS, 0.2% Triton X-100, and 500 mM NaCl) and eluted with 100 µL of elution buffer (50 mM ammonium bicarbonate [NH_4_HCO_3_], pH 8.2) containing 5 mM TCEP to release peptide fragments containing free thiol residues (corresponding to persulfidation sites). Iodoacetamide (IAA) was added to the eluted fraction at a final concentration of 20 mM to alkylate free thiols at room temperature in the dark for 1 h. Following drying and desalting, samples were vacuum concentrated, dissolved in 10 µL of 0.1% formic acid (FA), and subjected to mass spectrometry analysis [31,32].

### 2.13. LC-MS/MS Analysis and Database Search

Peptide samples were separated using an Easy nLC 1200 chromatography system (Thermo Scientific, San Jose, CA, USA) at a flow rate of 300 nL/min. Mobile phase A was 0.1% formic acid in water, and mobile phase B was 0.1% formic acid in 85% acetonitrile. Peptides were first loaded onto a trap column (100 μm × 20 mm, 5 μm, C18, Dr. Maisch GmbH, Ammerbuch, Germany) and then separated on an analytical column (75 μm × 150 mm, 3 μm, C18, Dr. Maisch GmbH, Ammerbuch, Germany) using the following gradient: 5–8% B from 0–2 min, 8–23% B from 2–90 min, 23–40% B from 90–100 min, 40–100% B from 100–108 min, and 100% B from 108–120 min.

The eluted peptides were analyzed on a Q-Exactive HF-X mass spectrometer (Thermo Scientific, San Jose, CA, USA) in positive ion mode over 120 min. Full MS scans were acquired in the range of 300–1800 *m*/*z* at a resolution of 60,000 (@200 *m*/*z*), with an AGC target of 1 × 10^6^ and a maximum injection time of 50 ms. For each full scan, the top 20 most intense precursor ions were selected for MS/MS fragmentation using higher-energy collision dissociation (HCD) at a normalized collision energy of 28%. MS2 spectra were collected at a resolution of 15,000 (@200 *m*/*z*), with an AGC target of 1 × 10^5^, a maximum injection time of 50 ms, and an isolation window of 1.6 Th.

Raw MS/MS data were searched using MaxQuant (version 1.6.15.0) against the UniProt database (uniprotkb-*Pseudomonas aeruginosa* PAO1). Target-decoy strategy was applied during database searching, with the decoy database generated by reversing the target sequences. Trypsin/P was set as the cleavage enzyme, allowing up to two missed cleavages. The minimum peptide length was 7 amino acids, and a maximum of five modifications per peptide was permitted. Precursor mass tolerance was 20 ppm for both first and main searches, and fragment ion mass tolerance was 20 ppm. Carbamidomethylation of cysteine was specified as a variable modification, which introduces a mass shift of +57.0215 Da. Site localization was performed using Spectronaut’s built-in PTM site-scoring algorithm, with a site probability of ≥0.75 as the cutoff for reliable localization. False discovery rates (FDRs) for protein, peptide, and PSM levels were all set below 1%.

Gene Ontology (GO) annotation was performed using the eggnog-mapper software (version 2.1.14) to extract GO identifiers from identified proteins based on the EggNOG database, followed by functional classification of proteins according to cellular components, molecular functions, and biological processes.

The mass spectrometry proteomics data have been deposited to the ProteomeXchange Consortium https://proteomecentral.proteomexchange.org (accessed on 27 May 2026) via the iProX partner repository with the dataset identifier PXD078215.

### 2.14. Enzyme Activity Determination of Dihydrolipoamide Dehydrogenase (LpdG)

Dihydrolipoamide dehydrogenase activity was measured spectrophotometrically by monitoring the reduction of NAD^+^ to NADH, coupled to the oxidation of dihydrolipoamide. The reaction was performed in a total volume of 1 mL containing 100 mM potassium phosphate buffer (pH 7.0), 2 mM NAD^+^, 0.1 mM MgCl_2_, 0.1 mM coenzyme A, 0.2 mM thiamine pyrophosphate, 1 mM MgCl_2_, 0.2 mM dithiothreitol (DTT), 5 mM L-valine, and 10 ng of purified protein. The reaction was initiated by adding 0.1 mM DL-lipoamide as the substrate.

The production of NADH was continuously monitored at 340 nm, while the consumption of NAD^+^ was monitored at 360 nm using a Synergy 2 Pro200 Multi-Detection Microplate Reader (Tecan, Männedorf, Switzerland). All measurements were performed at 37 °C over a period of 60 min, with readings taken every 30 s. Enzyme activity was calculated as nanomoles of NADH produced per minute per milligram of protein (nmol/min/mg). All assays were performed in triplicate, and data are presented as mean ± SEM.

## 3. Results

### 3.1. Sulfane Sulfur Generated by Sulfide: Quinone Oxidoreductase (SQR) Serves as a Key Mediator in the Oxidative Stress Defense of P. aeruginosa PAO1

**Figure 1:**
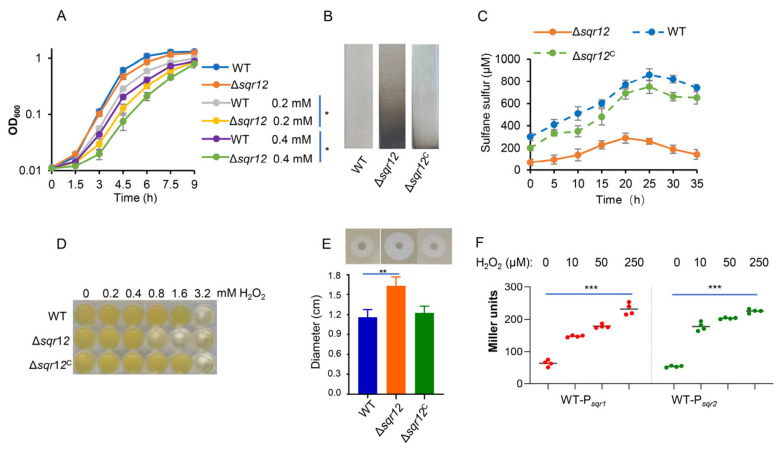
The effects of endogenous sulfane sulfur on the H_2_O_2_ sensitivity of *P. aeruginosa* PAO1. (**A**) Growth curves of the indicated strains in LB medium supplemented with various concentrations of H_2_O_2_. (**B**) H_2_S generation by the indicated mutants grown in LB medium was monitored using lead acetate strips under aerobic conditions. (**C**) Intracellular sulfane sulfur levels in the indicated strains. (**D**) MIC survival rate determination. (**E**) Disk diffusion assay for H_2_O_2_. Paper disks containing 10 µL of 0.1 M H_2_O_2_ were placed on the plates. (**F**) Effect of H_2_O_2_ on *sqr* promoter activity in aerobically grown WT cells. β-Galactosidase activities of the integrated *lac*Z reporters were determined and are presented as Miller units. All experiments were repeated independently at least three times. Data are shown as the mean ± SEM. For (**A**), statistical comparisons between the indicated strains were performed using two-way repeated-measures ANOVA with GraphPad Prism software (version 10.1.2). For (**E**,**F**), statistical comparisons between two groups were performed using an unpaired two-tailed Student’s *t*-test with GraphPad Prism software. Asterisks indicate statistically significant differences (* *p* < 0.05; ** *p* < 0.01; *** *p* < 0.001).

Two conserved pathways contribute to RSS biosynthesis in *P. aeruginosa* PAO1: the cysteine metabolic pathway, which generates limited sulfane sulfur from cysteine precursors, and the SQR-dependent route, which directly oxidizes H_2_S to yield sulfane sulfur. To identify the dominant RSS biosynthetic pathway, we constructed an in-frame double-deletion strain lacking two SQR genes (*PA2566* and *PA2345*), designated Δ*sqr12*. Although Δ*sqr12* displayed growth rate indistinguishable from that of the WT in aerobic LB medium (Figure 1A), lead acetate strip assays revealed that it accumulated and released detectable H_2_S after 12 h of cultivation, in contrast to the negligible release observed in the WT (Figure 1B). This finding demonstrates the role of SQR in converting endogenous H_2_S to sulfane sulfur, thereby limiting its volatilization. Consistent with this, cellular sulfane sulfur levels were markedly reduced in Δ*sqr12* cells compared with the WT during aerobic growth in LB, with the most pronounced decline occurring in the stationary phase (Figure 1C).

We next assessed the physiological impact of sulfane sulfur deficiency on oxidative stress resistance by comparing H_2_O_2_ sensitivity between the WT and Δ*sqr12* strains. Growth inhibition assays revealed that Δ*sqr12* exhibited impaired growth relative to the WT when exposed to ≥0.2 mM H_2_O_2_ (Figure 1A and Figure S2A). Consistent with this phenotype, the mutant showed a lower minimal inhibitory concentration (MIC) in liquid culture (Figure 1D) and generated inhibition zones that were 1.35-fold larger than those of the WT in disk diffusion assays (Figure 1E), collectively demonstrating the increased susceptibility of Δ*sqr12* to H_2_O_2_-induced stress.

Genetic complementation of Δ*sqr12* with a functional SQR expression construct (Δ*sqr12*^C^) fully restored H_2_O_2_ resistance to WT levels (Figure 1D,E and Figure S2A), confirming that the observed phenotype is specifically linked to sulfane sulfur biosynthesis. Notably, although the intracellular H_2_S levels in Δ*sqr12* were higher than those in the WT (Figure 1B), its oxidative stress resistance remained compromised, indicating that H_2_S itself cannot functionally substitute for sulfane sulfur in ROS protection. This notion was further supported by the 3.7- and 4.2-fold upregulation of the promoter activities of *sqr1* and *sqr2* upon exposure to 400 μM H_2_O_2_ (Figure 1F), illustrating a positive feedback loop between oxidative stress and sulfane sulfur biosynthesis. To quantitatively assess the role of SQRs in H_2_O_2_ detoxification, we complemented Δ*sqr12* with plasmid-encoded *sqr1* under IPTG-inducible control, which revealed a clear inverse correlation between SQR expression and inhibition zone diameter. At 0.15 mM IPTG, the complemented strain exhibited a 0.93 cm inhibition zone, smaller than the 1.16 cm zone observed for the WT (Figure S2B).

### 3.2. Sulfane Sulfur Mediates H_2_O_2_ Resistance Independent of Direct ROS Scavenging

**Figure 2:**
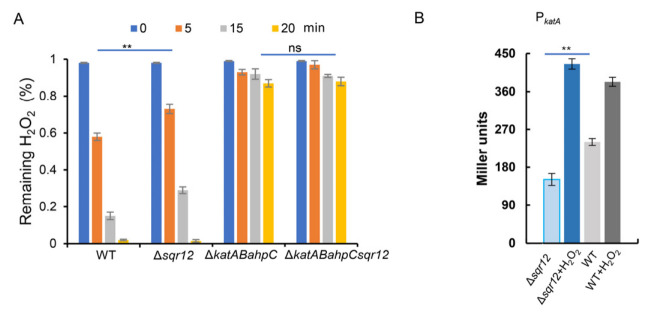
Impacts of sulfane sulfur on H_2_O_2_ scavenging capacity. (**A**) Degradation of 0.1 mM H_2_O_2_ by the indicated strains. The remaining H_2_O_2_ concentration was measured at the indicated time points. Relative H_2_O_2_ concentrations were calculated by normalizing each value to the initial concentration. (**B**) Expression of *katA* in WT and Δ*sqr* strains in response to induction with 0.4 mM H_2_O_2_. All experiments were repeated at least three times using independent biological replicates. Data are presented as the mean ± SEM. For (**A**), two-way ANOVA followed by Šídák’s multiple-comparisons test was used to compare the WT and Δ*sqr* groups. For (**B**), an unpaired two-tailed Student’s *t*-test was used. Asterisks indicate significant differences (ns, not significant, ** *p* < 0.01).

To determine whether sulfane sulfur contributes to oxidative stress defense by enhancing H_2_O_2_ scavenging capacity, mid-exponential-phase bacteria were challenged with 0.1 mM H_2_O_2_, and residual H_2_O_2_ levels were monitored over a 20-min period. WT cells exhibited greater H_2_O_2_ clearance capacity than Δ*sqr12* (Figure 2A), suggesting that sulfane sulfur may promote H_2_O_2_ detoxification either directly or indirectly by upregulating the expression of major H_2_O_2_ scavengers, including the catalases KatA and KatB and the alkyl hydroperoxide reductase AhpC, in PAO1.

We then constructed a catalase/peroxidase-deficient strain (Δ*katA*Δ*katB*Δ*ahpC*) in PAO1 and its sulfane sulfur-deficient derivative (Δ*katA*Δ*katB*Δ*ahpC*Δ*sqr12*) for H_2_O_2_ degradation assays (Figure 2A). The similar H_2_O_2_ degradation kinetics observed for Δ*katA*Δ*katB*Δ*ahpC* and Δ*katA*Δ*katB*Δ*ahpC*Δ*sqr12* indicate that the sulfane sulfur-enhanced H_2_O_2_ clearance capacity of WT cells does not depend on direct H_2_O_2_ scavenging by sulfane sulfur. Instead, the impaired H_2_O_2_ clearance capacity of Δ*sqr12* is likely attributable to reduced basal expression of major H_2_O_2_ scavengers. In Gram-negative bacteria, the expression of *katA*, *katB*, and *ahpC* is regulated by OxyR [33,34]. To dissect the effect of sulfane sulfur on OxyR regulation, we employed a *lac*Z reporter to monitor the expression level of the housekeeping catalase gene *katA* in WT and Δ*sqr12* strains. Under normal physiological conditions, when the intracellular H_2_O_2_ concentration is low, the key cysteine residues of OxyR exist in the free thiol (-SH) form. In this state, OxyR adopts its reduced conformation and lacks regulatory activity toward the *katA* promoter in Δ*sqr12* background. In contrast, WT cells exhibited significantly higher basal *katA* transcription (Figure 2B), suggesting partial activation of OxyR. This was further confirmed by detecting *katA* and *ahpC* expression in the chromosomal OxyR variants Δ*oxyR*/*oxyR*(C199A) and Δ*oxyR*/*oxyR*(C208A). Basal expression of *katA* and *ahpC* was markedly decreased in the C199A mutant, and the reduction was comparable to that observed in the Δ*sqr12* strain under normal physiological conditions (Figure S4). In contrast, basal expression of *katA* and *ahpC* in the C208A mutant was comparable to that observed in the WT strain under normal physiological conditions (Figure S4). Upon H_2_O_2_ treatment, comparable *katA* upregulation was observed in both Δ*sqr12* and WT strains (Figure 2B), indicating that oxidative activation overrides sulfane sulfur-mediated regulation.

Emerging evidence indicates that sulfane sulfur mediates bacterial signaling through the persulfidation of conserved cysteine residues in transcriptional regulators, thereby modulating their conformational dynamics, DNA-binding affinity, and regulatory output [15,26,27,35]. OxyR contains six cysteine residues, including two conserved redox-sensing cysteines, Cys199 and Cys208, which are known to mediate its response to ROS. Cys199 is initially oxidized by H_2_O_2_ to form Cys199-SOH. Subsequently, Cys208 participates in the formation of an intramolecular disulfide bond with Cys199, generating the activated form of OxyR. Both C199A and C208A chromosomal point mutants of *P. aeruginosa* failed to respond to H_2_O_2_ stress and exhibited severely impaired H_2_O_2_ resistance (Figure S4). These findings suggest that *Pa*OxyR is likely an in vivo target of endogenous sulfane sulfur and that its regulatory activity may be mediated by persulfidation of conserved redox-sensing cysteines.

### 3.3. Transcription Factor OxyR Is Endogenously Persulfidated in P. aeruginosa PAO1

**Figure 3:**
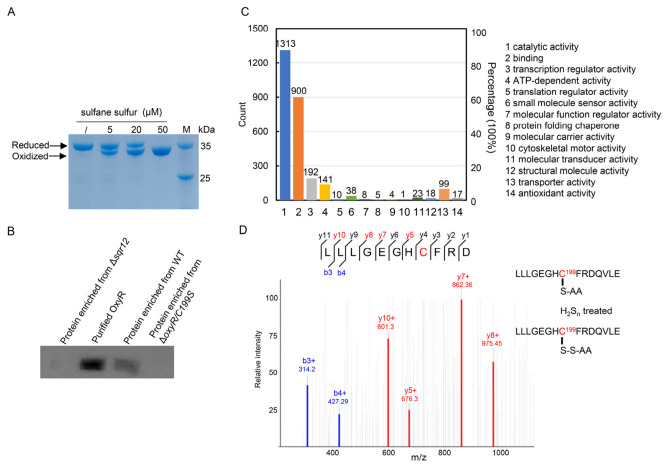
Identification of persulfidated protein targets involved in the oxidative stress response of *P. aeruginosa* PAO1. (**A**) Mobility shifts of H_2_S_n_-treated OxyR on SDS-PAGE. H_2_S_n_-treated and reduced OxyR proteins were precipitated with trichloroacetic acid (TCA), labeled with IAM, reduced with DTT, and then subjected to SDS-PAGE. (**B**) Biotin–maleimide-labeled proteins were affinity-captured using avidin beads from cell lysates of WT and Δ*sqr12* strains. Proteins containing persulfidated sites were specifically eluted with TCEP-containing buffer. OxyR was identified in the eluates by Western blotting. (**C**) GO enrichment analysis of persulfidated proteins in *P. aeruginosa* PAO1 identified by DIA-based quantitative proteomics. (**D**) Higher-energy collisional dissociation mass spectrum of the Cys199-containing peptide from OxyR.

To determine the redox status of OxyR regulated by sulfane sulfur, we expressed and purified C-terminally His_6_-tagged OxyR from *E. coli* (Figure S3) and treated the recombinant protein with sulfane sulfur (H_2_S_n_). Mobility shifts of H_2_S_n_-treated OxyR on SDS-PAGE confirmed the formation of persulfided OxyR species (Figure 3A). To establish the physiological relevance of this modification, we next performed persulfidation-specific enrichment from PAO1 cell lysates using maleimide–biotin labeling, followed by NeutrAvidin bead capture and TCEP-mediated elution of persulfidated proteins. Western blot analysis with an OxyR-specific antibody demonstrated a clear persulfidated OxyR signal in WT cells, whereas this signal was abolished in the sulfane sulfur-deficient Δ*sqr12* mutant (Figure 3B). This SQR-dependent persulfidation confirms that endogenous sulfane sulfur generated by Sqr1 and Sqr2 directly modifies OxyR in vivo.

To identify the conserved redox-sensing cysteine residue of OxyR that undergoes persulfidation in vivo in PAO1, we conducted a data-independent acquisition (DIA) mass spectrometry-based persulfidomic analysis, which identified 4751 persulfidated cysteine residues across 3160 distinct proteins (Supplementary Data S1). Compared with traditional data-dependent acquisition (DDA) mass spectrometry, DIA mass spectrometry unbiasedly fragments all precursor ions within defined isolation windows and records comprehensive fragment ion information, whereas DDA preferentially selects the most abundant precursor ions for MS/MS fragmentation. Therefore, DIA enables more comprehensive detection of low-abundance proteins, reduces missing values, and improves data completeness. Gene ontology (GO) enrichment analysis revealed the broad involvement of persulfidated proteins in core cellular processes, including catalytic activity, energy metabolism, transcriptional regulation, and, most notably, antioxidant activity (Figure 3C). These results indicate that several persulfidated proteins detected by persulfidomic analysis are likely to play key roles in the oxidative stress response of *P. aeruginosa* PAO1. Furthermore, our findings suggest that sulfane sulfur exerts its protective effects against ROS through the selective modification of key regulatory and enzymatic protein targets (Table 2). Notably, our persulfidomic profiling identified Cys199 as a prominent in vivo persulfidation site of OxyR in PAO1 (Figure 3D). This result was further supported by the loss of the persulfidated OxyR signal in the chromosomal OxyR C199A mutant (Figure 3B), suggesting a novel regulatory mechanism beyond classical H_2_O_2_-induced disulfide bond formation. This modification may stabilize a distinct single-cysteine-activated conformation of OxyR.

### 3.4. Sulfane Sulfur Fine-Tunes OxyR Regulatory Activity Through Redox-State Modulation

**Figure 4:**
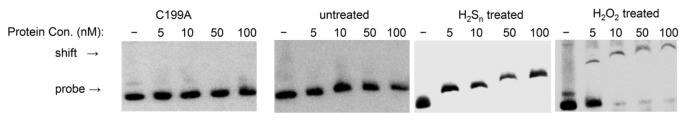
In vitro interaction of persulfidated OxyR with the *katA* promoter analyzed by EMSA. His_6_-tagged OxyR was purified from *E. coli* BL21 and pretreated with 50 µM H_2_S_n_ or 200 µM H_2_O_2_. The purified OxyR protein (5–100 nM) used for EMSA was pre-reduced with 1 mM dithiothreitol (DTT) for 30 min to ensure complete reduction and minimize air oxidation. Unreacted DTT was then removed using a desalting column. The reduced OxyR was subsequently treated with sulfane sulfur or H_2_O_2_ at the indicated concentrations for 30 min at room temperature to generate persulfidated or oxidized OxyR, respectively. Binding reactions containing a biotin-labeled *katA* promoter probe and increasing amounts of pretreated OxyR were incubated and resolved by native PAGE. Protein–DNA complexes were transferred to a nylon membrane, crosslinked, and detected by chemiluminescence.

To compare the differences in oligomerization state and promoter-binding capacity among distinct redox states of OxyR, recombinant C-terminally His_6_-tagged OxyR and its mutants were expressed in *E. coli* and purified by Ni-affinity chromatography for electrophoretic mobility shift assay (EMSA) using the *katA* promoter as a target. The results revealed that although reduced OxyR, H_2_S_n_-modified OxyR, and H_2_O_2_-oxidized OxyR all bound the *katA* promoter, their binding patterns and DNA–protein complex formation differed significantly. Control experiments with the C199A OxyR mutant confirmed the essential role of Cys199, as this variant completely abolished DNA-binding activity, establishing Cys199 as the primary sensor for both sulfane sulfur and H_2_O_2_ (Figure 4).

Notably, H_2_S_n_-modified OxyR exhibited an intermediate binding affinity, stronger than that of the reduced form but weaker than that of fully oxidized OxyR, and promoted the formation of intermediate-order oligomeric complexes. In contrast, H_2_O_2_-oxidized OxyR generated clear supershifted bands corresponding to higher-order oligomers, including tetramers, hexamers, and octamers, indicating enhanced cooperative DNA binding (Figure 4). Collectively, the oligomerization state of OxyR correlates with its transcriptional regulatory activity: reduced OxyR acts as a very weak DNA-binding form with minimal activity, persulfidated OxyR functions as a moderate DNA-binding partial activator, and oxidized OxyR serves as a strong DNA-binding full activator. WT cells maintain KatA at intermediate expression levels (Figure 2B), consistent with the partial activation state of persulfidated OxyR, which represents a functional intermediate between the fully reduced and fully oxidized forms.

### 3.5. Identification of the ROS Vulnerable Protein Protected by Sulfane Sulfur

Previous studies in eukaryotic systems have demonstrated that protein persulfidation can protect cysteine thiols in ROS-vulnerable proteins from irreversible oxidative damage [4,16,17,18]. To investigate whether sulfane sulfur performs a similar protective function in *P. aeruginosa*, we employed a transposon mutagenesis approach to identify suppressors of the oxidative stress sensitivity observed in the Δ*sqr12* mutant. Given that Δ*sqr12* cells exhibit reduced intracellular sulfane sulfur levels and, consequently, diminished protein persulfidation, we hypothesized that overexpression of potential sulfane sulfur target proteins might compensate for this protective deficit.

**Figure 5:**
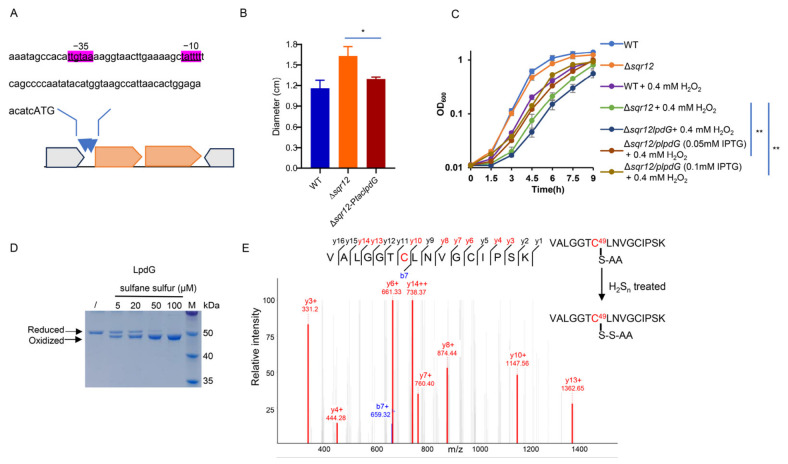
Screening for mutants with enhanced Δ*sqr12* viability by transposon mutagenesis. (**A**) Schematic overview of the transposon screening strategy. The blue arrow indicates that the transposon insertion sequence contains a strong promoter, enabling overexpression of the downstream gene at the insertion site. (**B**) Susceptibility of the indicated strains to H_2_O_2_ as determined by a disk diffusion assay. (**C**) Growth of the indicated strains in LB medium in the presence of H_2_O_2_. (**D**) Mobility shifts of persulfidated LpdG on SDS-PAGE. H_2_S_n_-treated and reduced LpdG proteins were precipitated with trichloroacetic acid (TCA), labeled with IAM, reduced with DTT, and then subjected to SDS-PAGE. (**E**) Persulfidation of the reactive Cys49 residue of LpdG was detected by higher-energy collisional dissociation mass spectrometry. All experiments were repeated at least three times as independent biological replicates. Data are presented as the mean ± SEM. For (**B**), an unpaired two-tailed Student’s *t*-test was used. For (**C**), statistical comparisons between the indicated strains were performed using two-way repeated-measures ANOVA. Asterisks indicate significant differences (* *p* < 0.05; ** *p* < 0.01).

We therefore constructed a transposon library in Δ*sqr12* using a mobile element containing a strong constitutive promoter (Figure 5A). Random insertion of this promoter enabled transcriptional activation of downstream genes, potentially overcoming oxidative damage to proteins that would normally be protected by sulfane sulfur-mediated persulfidation. Following conjugation between Δ*sqr12* and *E. coli* WM3064 carrying the transposon vector, the resulting library was challenged with different concentrations of H_2_O_2_ (0.5, 1, and 2 mM) for 20 min before plating on gentamicin-containing selective medium.

Primary screening identified approximately 300 mutants with significantly enhanced H_2_O_2_ resistance, from which we selected eight colonies exhibiting stable and robust growth phenotypes for further characterization. Arbitrary PCR mapping revealed that the promoter region of PA1857 (*lpdG*) was targeted in four independent insertions, suggesting strong selection pressure for *lpdG* overexpression in the Δ*sqr12* background. *lpdG* encodes a redox active disulfide oxidoreductase containing catalytic cysteine residues and functions as an essential component of both the pyruvate dehydrogenase and 2-oxoglutarate dehydrogenase complexes [36]. Previous studies have shown that *lpdG* mutations result in markedly reduced NADH-dependent dihydrolipoyl dehydrogenase activity, which is involved in 2-oxoglutarate and pyruvate metabolism.

Validation experiments confirmed that plasmid-based overexpression of *lpdG* partially restored H_2_O_2_ resistance in Δ*sqr12* (Figure 5B), indicating that LpdG is closely associated with the H_2_O_2_ sensitivity caused by sulfane sulfur deficiency. To further characterize this relationship, we constructed Δ*sqr12*Δ*lpdG* and Δ*sqr12*-P*tac*-*lpdG* strains for phenotypic analysis. Growth curves of Δ*sqr12*Δ*lpdG* and Δ*sqr12*-P*tac*-*lpdG* were monitored in the presence of 0.4 mM H_2_O_2_. Under this condition, the growth of Δ*sqr12*Δ*lpdG* and Δ*sqr12* was inhibited by oxidative stress. However, overexpression of *lpdG* partially alleviated the growth inhibition of Δ*sqr12*, implying that *lpdG* depletion impairs the ability of cells to defend against H_2_O_2_ stress (Figure 5C).

To test whether LpdG undergoes sulfane sulfur-mediated modification, recombinant LpdG–6× His was treated with increasing concentrations of sulfane sulfur, blocked with iodoacetamide, reduced with DTT, and analyzed by SDS-PAGE (Figure 5D). The progressive downward band shift observed with increasing sulfane sulfur is consistent with DTT-mediated reduction of the persulfidated IAM adduct (R-SS-IAM) to a faster-migrating free thiol (R-SH), indicating that specific cysteine residues in LpdG undergo persulfidation. Furthermore, mass spectrometry analysis identified Cys49 of LpdG as a persulfidation site (Figure 5E).

### 3.6. Persulfidation-Mediated Protection of LpdG Against Irreversible Oxidative Inactivation

**Figure 6:**
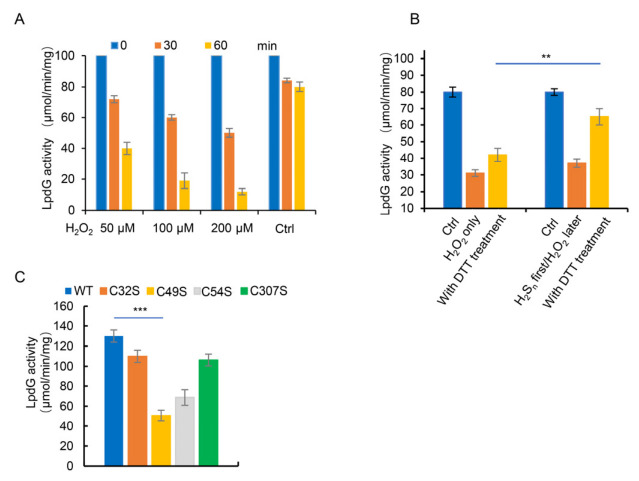
Reversible inhibition of LpdG activity upon H_2_S_n_ and H_2_O_2_ treatment. (**A**) Recombinant LpdG (20 μM) was incubated with 50, 100, or 200 μM H_2_O_2_ for 30 or 60 min at 37 °C. The enzymatic activity of LpdG was measured by spectrophotometric assays using NAD^+^ as the substrate. (**B**) Recombinant LpdG (20 μM) was incubated with or without 50 μM H_2_S_n_ for 30 min, followed by treatment with 200 μM H_2_O_2_ for 40 min at 25 °C. Samples were then treated with or without 2 mM DTT for 30 min at 37 °C. Lipoamide dehydrogenase activity of LpdG was measured by spectrophotometric assays using NAD^+^ as the substrate. (**C**) Lipoamide dehydrogenase activities of LpdG mutant proteins were measured. All experiments were repeated at least three times independently as biological replicates. Data are presented as the mean ± SEM. For panels (**B**,**C**), an unpaired two-tailed Student’s *t*-test was used. Asterisks indicate statistically significant differences (** *p* < 0.01; *** *p* < 0.001).

The observed growth restoration in Δ*sqr12* upon *lpdG* overexpression strongly suggested that this enzyme represents a ROS-sensitive target requiring protection by sulfane sulfur. To elucidate the molecular mechanism underlying this protection, we systematically investigated how persulfidation modulates the susceptibility of LpdG to oxidative inactivation. Initial kinetic analyses revealed concentration-dependent inhibition of LpdG activity upon H_2_O_2_ exposure, with 50 μM H_2_O_2_ reducing LpdG activity by 59% after 60 min of incubation, whereas higher H_2_O_2_ concentrations caused more severe activity loss, reaching up to 88% (Figure 6A).

We next tested whether preconditioning LpdG with sulfane sulfur could mitigate H_2_O_2_-induced oxidative damage in vitro. Preincubation of LpdG with 50 μM H_2_S_n_ for 30 min prior to challenge with 200 μM H_2_O_2_ resulted in marked preservation of enzymatic activity (Figure 6B). Reduction-rescue experiments showed that DTT restored only 11% of activity in H_2_O_2_-treated samples but achieved significantly greater recovery in enzymes that had been preconditioned with H_2_S_n_ prior to oxidation (Figure 6B). This enhanced reversibility indicates that persulfidation establishes a protected redox state, allowing more efficient enzymatic reactivation following oxidative damage.

To identify the critical cysteine residues involved in this protective mechanism, we generated individual serine substitutions for all four cysteine residues of LpdG (Cys32, Cys49, Cys54, and Cys307). Functional characterization revealed that mutations at Cys49 or Cys54, but not at Cys32 or Cys307, impaired catalytic activity (Figure 6C), identifying these residues as essential for LpdG enzymatic function and potentially important for persulfidation-mediated protection.

**Figure 7:**
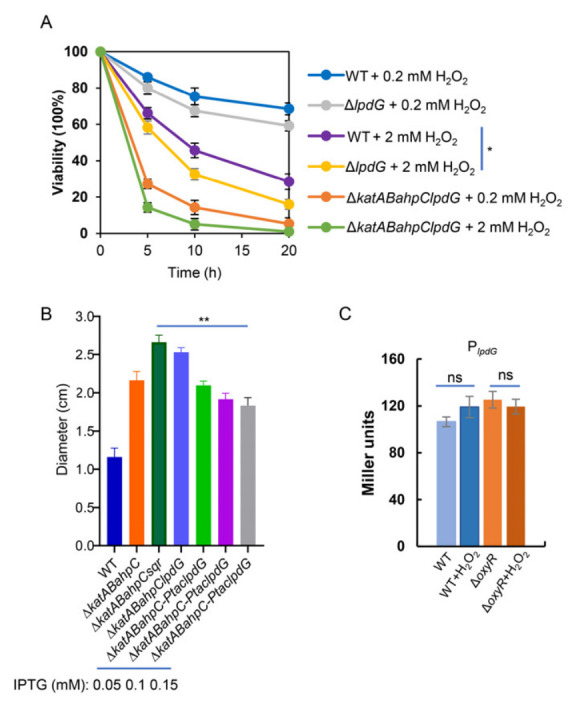
Role of LpdG in H_2_O_2_ resistance. (**A**) Survival assay. H_2_O_2_ was added to mid-log-phase cultures at the indicated final concentrations. After 5, 10, and 20 min, samples were appropriately diluted and plated on LB agar plates. Colony counts were performed after 24 h of incubation. All experiments were performed three times, and similar results were obtained. (**B**) Disk diffusion assay assessing susceptibility to H_2_O_2_. (**C**) Effect of H_2_O_2_ on *lpdG* promoter activity in WT and Δ*oxyR* strains grown aerobically. All experiments were repeated at least three times independently as biological replicates. Data are shown as the mean ± SEM. For panel (**A**), statistical comparisons between the indicated strains were performed using two-way ANOVA. For panels (**B**,**C**), an unpaired two-tailed Student’s *t*-test was used to compare the two indicated groups. Asterisks indicate statistically significant differences (ns, not significant, * *p* < 0.05; ** *p* < 0.01).

The physiological significance of LpdG protection was assessed through comprehensive viability assays. Although 0.2 mM H_2_O_2_ had minimal effects on survival in both WT and Δ*lpdG* strains, exposure to 2 mM H_2_O_2_ revealed marked differences: Δ*lpdG* exhibited pronounced survival defects, with viability decreasing to 58.3%, 32.6%, and 16% of the initial level at 5, 10, and 20 min, respectively (Figure 7A). In contrast, WT cells showed only a moderate reduction in viability, decreasing to 86%, 75.4%, and 68.5% of the initial level at the same time points. The Δ*katAB*Δ*ahpC*Δ*lpdG* mutant showed the greatest reduction in viability (to below 30% after 5 min) under both H_2_O_2_ concentrations, underscoring the essential role of LpdG when canonical detoxification systems are compromised. The H_2_O_2_ resistance of the chromosomal Δ*lpdG*/*lpdG*(C49S) mutant was indistinguishable from that of the Δ*lpdG* strain, further supporting the critical role of Cys49 in LpdG function under oxidative stress (Figure S5).

Disk diffusion assays corroborated these findings, with Δ*katA*Δ*katB*Δ*ahpC*Δ*lpdG* generating an inhibition zone of 2.6 cm, comparable to that of Δ*katA*Δ*katB*Δ*ahpC*Δ*sqr*. Notably, *lpdG* overexpression in Δ*katA*Δ*katB*Δ*ahpC* enhanced H_2_O_2_ resistance in an IPTG concentration-dependent manner (Figure 7B). Promoter activity assays demonstrated that *lpdG* expression was not induced by H_2_O_2_ in either WT or Δ*oxyR* backgrounds, excluding regulation through the OxyR pathway (Figure 7C). Furthermore, H_2_O_2_ consumption assays showed comparable degradation kinetics between WT and Δ*lpdG* strains (Figure S6), confirming that LpdG does not contribute directly to H_2_O_2_ scavenging.

In summary, these findings indicate that LpdG is a ROS-sensitive metabolic enzyme and that its protection through sulfane sulfur-mediated persulfidation represents a key antioxidant strategy. This mechanism operates independently of direct H_2_O_2_ scavenging and instead preserves metabolic enzyme function under oxidative stress conditions, particularly when primary detoxification systems are compromised.

## 4. Discussion

Bacterial survival under oxidative stress conditions is continuously challenged by ROS-induced damage to cellular macromolecules, particularly chromosomal DNA and functional proteins [37,38]. To counter these threats, bacteria have evolved sophisticated defense systems comprising ROS-scavenging enzymes, damage-repair proteins, and responsive regulatory networks, which collectively minimize damage to sensitive cellular targets and promote the rapid recovery of damaged proteins [4,39].

Our study revealed that sulfane sulfur metabolism is intrinsically linked to the oxidative stress defense capacity of *P. aeruginosa*. The fate of endogenous H_2_S depends on the metabolic capacity of the bacterium. In organisms possessing SQR, including *P. aeruginosa*, H_2_S is efficiently captured and oxidized to generate sulfane sulfur under aerobic conditions. In contrast, bacteria lacking SQR, such as *E. coli*, release H_2_S into the gas phase [21,22,23,40,41].

We provide evidence demonstrating that SQR serves as the primary source of cellular sulfane sulfur in *P. aeruginosa* PAO1. SSP4-based fluorescence assays showed markedly reduced sulfane sulfur levels in the SQR-deficient mutant Δ*sqr12* (Figure 1C), which correlated with increased sensitivity to oxidative stress compared with WT cells (Figure 1A,D,E). These observations prompted further investigation into the mechanistic basis of sulfane sulfur-mediated protection against oxidative stress.

Given the dual nucleophilic and electrophilic reactivity of sulfane sulfur [42,43,44], we first examined its potential role as a direct H_2_O_2_ scavenger. However, in strains lacking major peroxide-scavenging enzymes, sulfane sulfur deficiency did not affect H_2_O_2_ clearance rates, indicating that direct peroxide quenching is unlikely to represent its primary protective mechanism. This finding is consistent with the chemical properties of sulfane sulfur, which predominantly exists in a deprotonated form intracellularly, thereby favoring thiol-based protein modification over direct ROS quenching.

The electrophilic nature of deprotonated sulfane sulfur facilitates protein persulfidation, a modification that appears to constitute a fundamental regulatory mechanism in the bacterial oxidative stress response. Our persulfidation proteomic analysis revealed widespread protein persulfidation in *P. aeruginosa*, resembling patterns observed in *Staphylococcus aureus* [45] and *Enterococcus faecalis* [46]. Notably, many antioxidant enzymes and the central ROS-sensing regulator OxyR underwent persulfidation in vivo in *P. aeruginosa*. This sulfane sulfur-mediated persulfidation mechanism helps resolve the paradox between the limited kinetic efficiency of H_2_S as a direct ROS scavenger, owing to competition with abundant cellular thiols [10,11], and its significant physiological role in oxidative stress protection. Rather than directly quenching ROS, sulfane sulfur appears to modulate cellular defense capacity through protein modifications that enhance resilience to oxidative stress.

OxyR, a LysR-type transcriptional regulator (LTTR) conserved in Gram-negative bacteria, functions as a primary sensor of intracellular H_2_O_2_ levels and coordinates oxidative stress responses [47,48,49]. Our investigation provides compelling evidence that cellular sulfane sulfur serves as an endogenous modulator of OxyR redox status and regulatory activity in *P. aeruginosa*.

**Figure 8:**
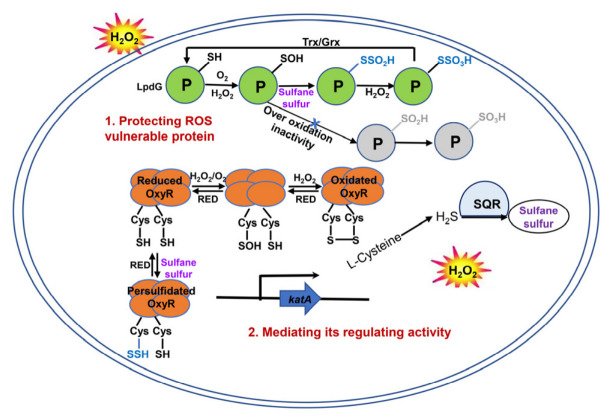
Schematic diagram of endogenous sulfane sulfur mediates the oxidative stress response process in *P. aeruginosa*.

We initially observed that basal *katA* expression in WT PAO1 was significantly higher than that in the Δ*sqr12* mutant, suggesting partial constitutive activation of OxyR in sulfane sulfur-replete cells. This phenomenon appears to arise from persulfidation at Cys199, which stabilizes an intermediate redox state distinct from both the fully reduced and fully oxidized forms. EMSA confirmed that this modified OxyR exhibits altered DNA-binding characteristics, with persulfidated OxyR showing reduced affinity for target promoters compared with the fully oxidized form. This intermediate activation state enables graded transcriptional responses that may optimize resource allocation during stress adaptation (Figure 8).

Proteomic and western blot analyses confirmed that Cys199 undergoes persulfidation to form Cys199-SSH in vivo, with identical modifications observed in vitro following H_2_S_n_ treatment. This PTM places OxyR within a growing class of redox-sensitive regulators that utilize cysteine modifications beyond simple disulfide bond formation. Previous studies have identified various oxidative modifications, including S-hydroxylation (S-OH), S-nitrosylation (SNO), and S-glutathionylation (S-SG), at the equivalent cysteine in EcOxyR [50,51], suggesting evolutionary conservation of this regulatory plasticity. Notably, sulfane sulfur-mediated OxyR modification represents a bifunctional adaptation: although originally characterized in the H_2_S_n_ stress response [28], our work demonstrates its relevance to H_2_O_2_ resistance. Since OxyR is widely distributed in both aerobic and anaerobic bacteria, the OxyR-regulated network may represent a conserved mechanism that bacteria employ when confronting endogenous and/or exogenous oxidative stress.

Our findings also reveal a fundamental cytoprotective mechanism whereby protein persulfidation safeguards redox-sensitive cysteine residues against irreversible oxidative damage. Unlike eukaryotic systems, in which polyunsaturated lipids are major ROS targets, bacterial oxidative injury predominantly affects proteins because bacteria contain relatively few lipid peroxidation substrates [20,52]. Cysteine residues are particularly vulnerable targets because of their nucleophilic thiol groups, which readily undergo overoxidation to form irreversible sulfonic acids under oxidative conditions (Figure 8).

We identified LpdG, a dihydrolipoamide dehydrogenase, as a representative ROS-vulnerable protein protected through persulfidation. The conserved Cys49 residue, which together with Cys54 constitutes the redox-active center of LpdG, undergoes selective persulfidation in vivo. Functional assays demonstrated that H_2_O_2_ exposure caused progressive activity loss, whereas pretreatment with H_2_S_n_ preserved enzymatic function through reversible persulfidation. Mass spectrometry confirmed Cys49 as the primary persulfidation site, with the modified peptides showing characteristic mass shifts.

Genetic evidence supports the critical role of LpdG in oxidative stress survival. Transposon mutagenesis identified *lpdG* overexpression as a suppressor of the H_2_O_2_ sensitivity of Δ*sqr12*, with promoter insertions enhancing bacterial viability. Subsequent validation showed that Δ*katA*Δ*katB*Δ*ahpC*Δ*sqr12* strains exhibited pronounced survival defects. Conversely, *lpdG* overexpression in sulfane sulfur-deficient backgrounds restored H_2_O_2_ resistance in a dose-dependent manner, confirming LpdG as a key protective target.

We propose a potential mechanism whereby persulfidation-based thiol protection in *P. aeruginosa* PAO1 accounts for the protective effects of sulfane sulfur against oxidative stress. Our experiments showed that persulfidation of LpdG may protect this enzyme from irreversible loss of activity caused by overoxidation. As cysteine residues in metabolic enzymes often serve as catalytic nucleophiles [20,53], their irreversible oxidation can permanently inactivate essential metabolic pathways. Our data suggest that constitutive persulfidation creates a protective “sacrificial layer” that is preferentially oxidized, thereby sparing catalytic cysteine residues from permanent damage. Protein persulfidation may therefore serve primarily as a preemptive mechanism rather than a reactive defense. According to this model, a basal level of persulfidation provides a built-in reservoir: once an oxidative event occurs, these pre-persulfidated proteins can be rapidly reactivated to facilitate cellular recovery. In contrast, if persulfidation were triggered only after the onset of stress, it would likely be too late to protect critical thiols, because the thiols most essential for cellular function are also among the most reactive toward H_2_O_2_ and would become irreversibly oxidized before they could be protectively persulfidated.

This strategy complements classical H_2_O_2_-scavenging systems by protecting the metabolic infrastructure against oxidative collapse. Our work establishes that protein persulfidation represents an evolutionarily conserved protective mechanism that parallels analogous systems in mammalian cells [16,17,18]. By integrating persulfidation mapping with genetic and biochemical validation, we demonstrate how sulfane sulfur mediates oxidative stress protection through the targeted safeguarding of metabolic enzymes, revealing a sophisticated layer of bacterial stress adaptation beyond conventional antioxidant systems (Figure 8).

## Figures and Tables

**Table 1:** Strains and plasmids used in this study.

Strain or Plasmid	Description	Source/Reference
Strains		
*E. coli*		
DH5α	Host strain for plasmids	[9]
WM3064	Donor strain for conjugation; Δ*dapA*	[9]
BL21	Recombinant protein expression host strain	[9]
*P. aeruginosa*		
PAO1	Wild type	ATCC 15692 (Biobw, Beijing, China)
PA2566-2345	Δ*sqr12* derived from PAO1	This study
PA5344	Δ*oxyR* derived from PAO1	This study
PA1857	Δ*lpdG* derived from PAO1	This study
PA4236-4613-0139	Δ*katABahpC* derived from PAO1	This study
PA4236-4613-0139-2566-2345	Δ*katABahpCsqr12* derived from PAO1	This study
PA2566-2345-1857	Δ*sqr12lpdG* derived from PAO1	This study
Δ*oxyR*/*oxyR* (C199A)	Point mutation for Residue Cys 199 replaced by Ala	This study
Δ*oxyR*/*oxyR* (C208A)	Point mutation for Residue Cys 208 replaced by Ala	This study
Δ*lpdG*/*lpdG* (C49S)	Point mutation for Residue Cys 49 replaced by Ser	This study
Plasmids		
pHGM01	Ap ^r^, Gm ^r^, CM ^r^, suicide vector	[7]
PHGE-P*tac*	Broad-host IPTG-inducible expression vector	[7]
pHGEI01	Integrative *lacZ* reporter vector	[7]
pFAC	Gm ^r^, vector containing transposable sequence	[7]
pET 28a	Recombinant protein expression vector	[9]
PHGE-P*tac*-*sqr1*	Vector for inducible expression of *sqr1*	This study
PHGE-P*tac*-*oxyR*	Vector for inducible expression of *oxyR*	This study
PHGE-P*tac*-*lpdG*	Vector for inducible expression of *lpdG*	This study
Plasmids		
PHGEI-P_ahpC_-*lac*Z	Vector for measuring P_ahpC_ activity	This study
PHGEI-P_katA_-*lac*Z	Vector for measuring P_katA_ activity	This study
PHGEI-P_sqr1_-*lac*Z	Vector for measuring P_sqr1_ activity	This study
PHGEI-P_sqr2_-*lac*Z	Vector for measuring P_sqr2_ activity	This study
PHGEI-P_lpdG_-*lac*Z	Vector for measuring P_lpdG_ activity	This study

^r^ indicates resistance.

**Table 2:** A summary of MS identified persulfidated proteins involved in antioxidant pathway of *P. aeruginosa* PAO1.

Accession	Gene	Description	Modified Sites	OxyR Regulon
O52762	*katA*	catalase A	IPVNAARCPHQVYHR	Yes
P23189	*gor*	glutathione reductase	GRVLEADCVFYATGR	Yes
P57668	*tpx*	thiol peroxidase	PFAQKRFCGAEGLEN, PSVDTPTCATSVRKF	Yes
Q9HVT5	PA4486	peroxiredoxin activity	RGALNNGCTVEEIRE, AALTALKCPQELKGH	
Q9HY81	PA3529	peroxidase	HEEHGEVCPANWKKG	
Q9I017	PA2826	glutathione peroxidase	DSLLSIPCTTIKGEQ, LVVLGFPCNQFGKQE, VVNTASKCGFTPQYQ	
Q9I078	PA2765	Dyp-type peroxidase C-terminal domain-containing protein	ISGSYFWCPPMADGR	
Q9I0M2	PA2616	*trxB*	ITSAGAGCMAALDAE, GVSACATCDGFFYRN, MGKGVSACATCDGFF, GVSACATCDGFFYRN, FYRNQVVCVVGGGNT	
Q9I1W8	PA2147	*katE*	WRPKSGTCSLVWDEA	Yes
Q9I2G4	PA1940	catalase core domain-containing protein	SIDRTPACYAFQVQR	
Q9I457	PA1287	glutathione peroxidase	SKESIDLCQRYAGKP, TAETAKICYGNYGVT	
Q9I4W5	PA1008	thioredoxin-dependent peroxiredoxin	PKDSTPGCTTEGQGF, SDKDEAVCQLFDVIK	
Q9I592	PA0849	thioredoxin reductase	GDNGTYTCDALIVAT	
Q9I5A2	PA0838	glutathione peroxidase	VVNVASKCGLTPQYA	
Q9I6M1	PA0269	carboxymuconolactone decarboxylase-like domain-containing protein	EQRLQALCVWQETPY	
Q9I6Z2	*ahpF*	alkyl hydroperoxide reductase subunit F	GVCFCPHCDGPLFKG, KAKGVCFCPHCDGPL, EYKAKGVCFCPHCDG, GVCFCPHCDGPLFKG, KAKGVCFCPHCDGPL	Yes
Q9I6Z3	*ahpC*	alkyl hydroperoxide reductase subunit C	AAHPGEVCPAKWKEG	Yes
Q9HTL4	*oxyR*	DNA-binding transcription factor of response to reactive oxygen species	LLLGEGHCFRDQVLE	Yes

## Data Availability

The original contributions presented in this study are included in the article/Supplementary Material. Further inquiries can be directed to the corresponding author.

## Supplementary Materials

The following supporting information can be downloaded at: https://www.mdpi.com/article/10.3390/antiox15060696/s1, Figure S1: Conservative mechanisms of protein oxidative damage and persulfidated modification protecting proteins; Figure S2: (A) Growth curves of the indicated strains in LB medium with 0.2 mM and 0.4 mM H_2_O_2_ added under aerobic conditions. (B) Disk diffusion assay for H_2_O_2_. Paper disks containing 10 µL of 0.1 M H_2_O_2_ were placed on the plate. Production of Sqr1 at different levels was induced by IPTG. All experiments were repeated at least three times (biological replicates). Data are presented as mean ± SEM. For (A) statistical comparisons between the indicated stains were performed using two-way ANOVA. For (B), unpaired two-tailed Student’s *t*-test was used. Asterisks indicate significant differences (ns, not significant; ** *p* < 0.01); Figure S3: SDS-PAGE of the purified C-terminally His_6_-tagged OxyR from *E. coli*; Figure S4: (A) Expression of the *katA* in WT and point mutant strains in response to 0.4 mM H_2_O_2_ induction. (B) Expression of the *ahpC* in WT and point mutant strains in response to 0.4 mM H_2_O_2_ induction. (C) Susceptibility of the indicated strains to H_2_O_2_ by disk diffusion assay. All experiments were repeated at least three times (biological replicates). Data are presented as mean ± SEM. Unpaired two-tailed Student’s *t*-test was used. Asterisks indicate significant differences (ns, not significant; ** *p* < 0.01; *** *p* < 0.001); Figure S5: Survival assay. The H_2_O_2_ was added to mid-log cultures to the final concentrations as indicated. After 5, 10 and 20 min, the samples were diluted appropriately and plated on LB plates. Colony counts were performed 24 h later. All experiments were repeated at least three times (biological replicates). Data are presented as mean ± SEM. Statistical comparisons between the indicated strains were performed using two-way ANOVA (ns, not significant); Figure S6: Degradation of 0.1 mM H_2_O_2_ by the indicated strains. The remaining H_2_O_2_ was measured at the indicated times. Relative concentrations of H_2_O_2_ were calculated by normalization of each value to the initial concentration. All experiments were repeated at least three times (biological replicates). Statistical comparisons between the indicated stains were performed using two-way ANOVA (ns, not significant); Table S1: The primers used in this study. Supplementary Data S1: A summary of MS identified persulfidated proteins.
