# Supplementary material for: Endogenous Sulfane Sulfur Mediates the Oxidative Stress Response Process in Pseudomonas aeruginosa
Source: Antioxidants (Basel). 2026 May 31;15(6):696. doi: 10.3390/antiox15060696 (PMC13295329; doi:10.3390/antiox15060696)
Supplement: Supplementary file 1 [file antioxidants-15-00696-s001.zip › supply information final.pptx]

## Slide 1
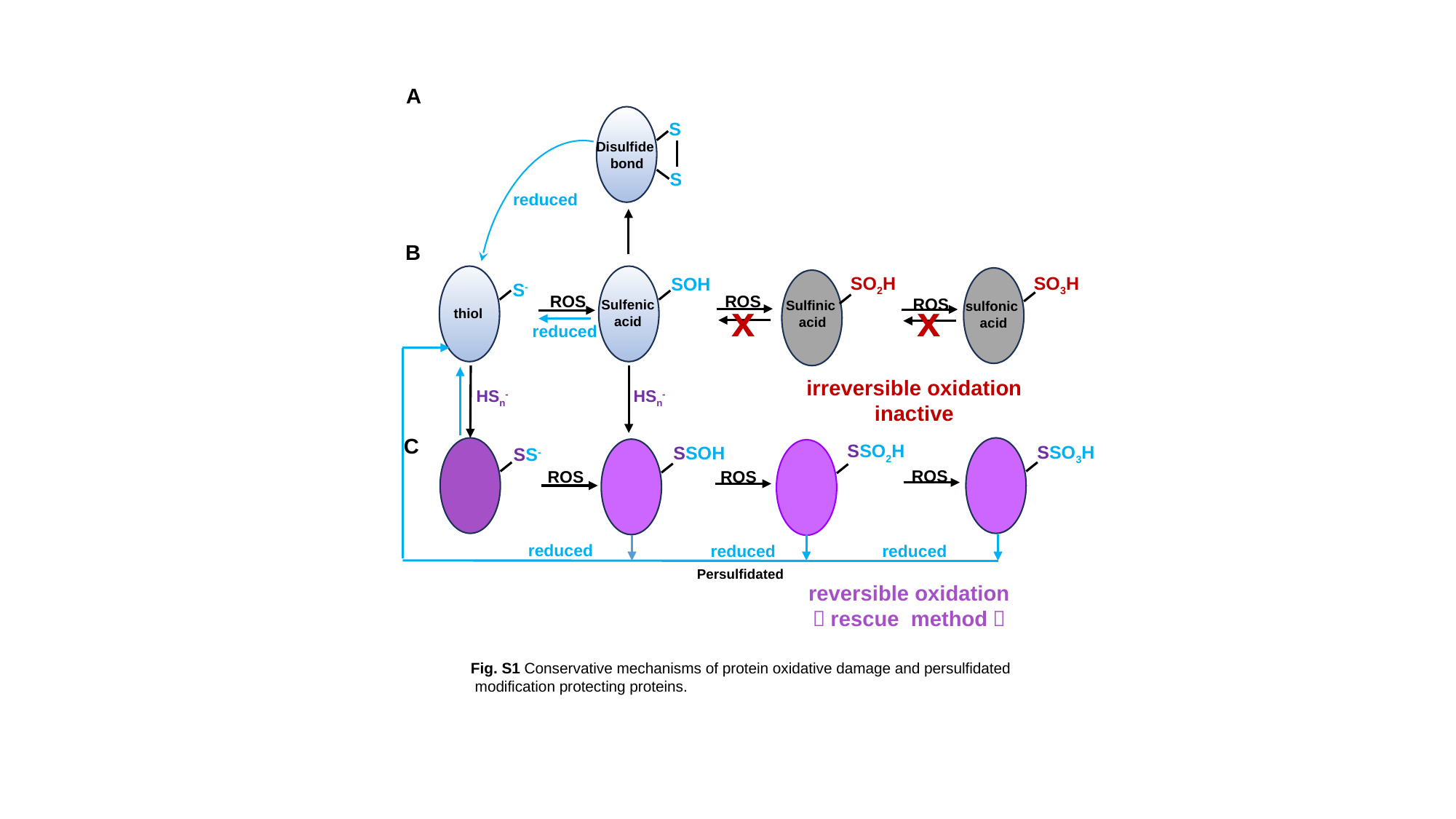

A
S
S
Disulfide
bond
reduced
B
ROS
SO2H
SO3H
SOH
S-
ROS
ROS
x
x
Sulfenic
acid
Sulfinic
acid
sulfonic
acid
thiol
reduced
irreversible oxidation
inactive
HSn-
HSn-
C
SSO2H
SSO3H
SSOH
SS-
ROS
ROS
ROS
reduced
reduced
reduced
Persulfidated
reversible oxidation
（rescue method）
Fig. S1 Conservative mechanisms of protein oxidative damage and persulfidated
 modification protecting proteins.

## Slide 2
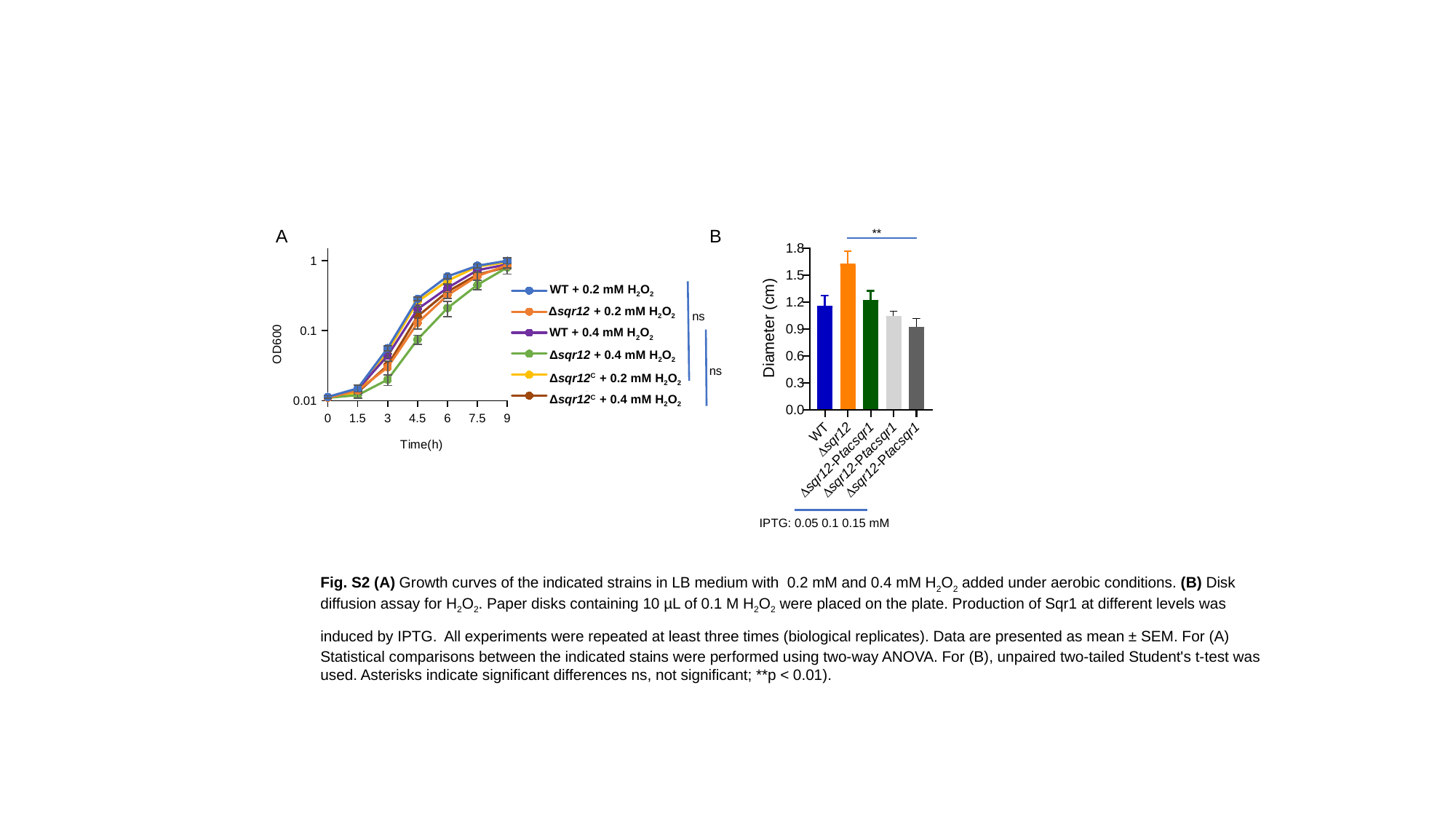

A
B
**
### Chart
| Category | wt H | sqr12 H | WT H2 | sqr H2 | sqr c-1 | sqr c-2 |
|---|---|---|---|---|---|---|WT + 0.2 mM H2O2
Δsqr12 + 0.2 mM H2O2
ns
WT + 0.4 mM H2O2
Δsqr12 + 0.4 mM H2O2
ns
Δsqr12C + 0.2 mM H2O2
Δsqr12C + 0.4 mM H2O2
IPTG: 0.05 0.1 0.15 mM
Fig. S2 (A) Growth curves of the indicated strains in LB medium with 0.2 mM and 0.4 mM H2O2 added under aerobic conditions. (B) Disk diffusion assay for H2O2. Paper disks containing 10 µL of 0.1 M H2O2 were placed on the plate. Production of Sqr1 at different levels was induced by IPTG. All experiments were repeated at least three times (biological replicates). Data are presented as mean ± SEM. For (A) Statistical comparisons between the indicated stains were performed using two‑way ANOVA. For (B), unpaired two‑tailed Student's t-test was used. Asterisks indicate significant differences ns, not significant; **p < 0.01).

## Slide 3
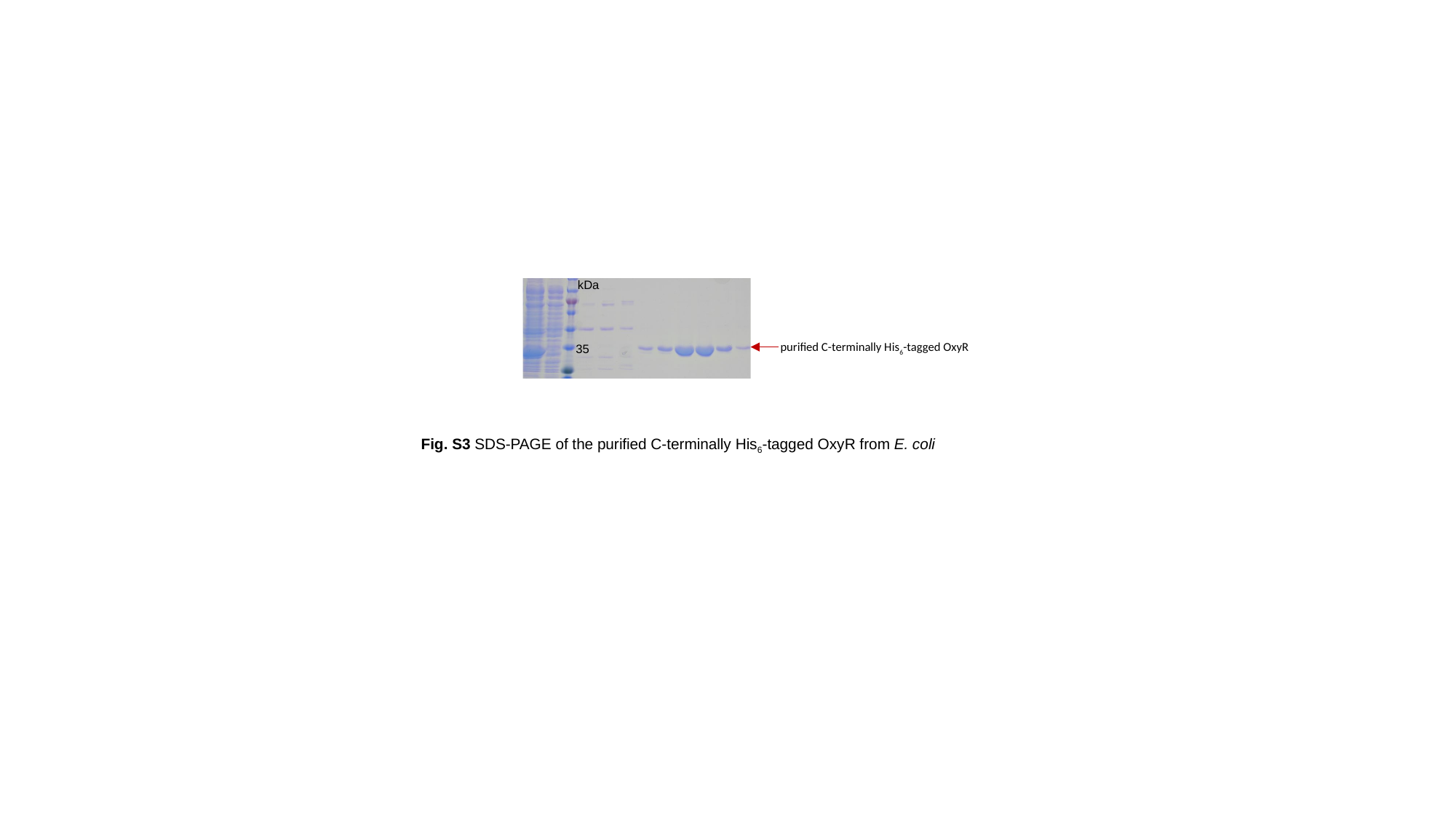

kDa
purified C-terminally His6-tagged OxyR
35
Fig. S3 SDS-PAGE of the purified C-terminally His6-tagged OxyR from E. coli

## Slide 4
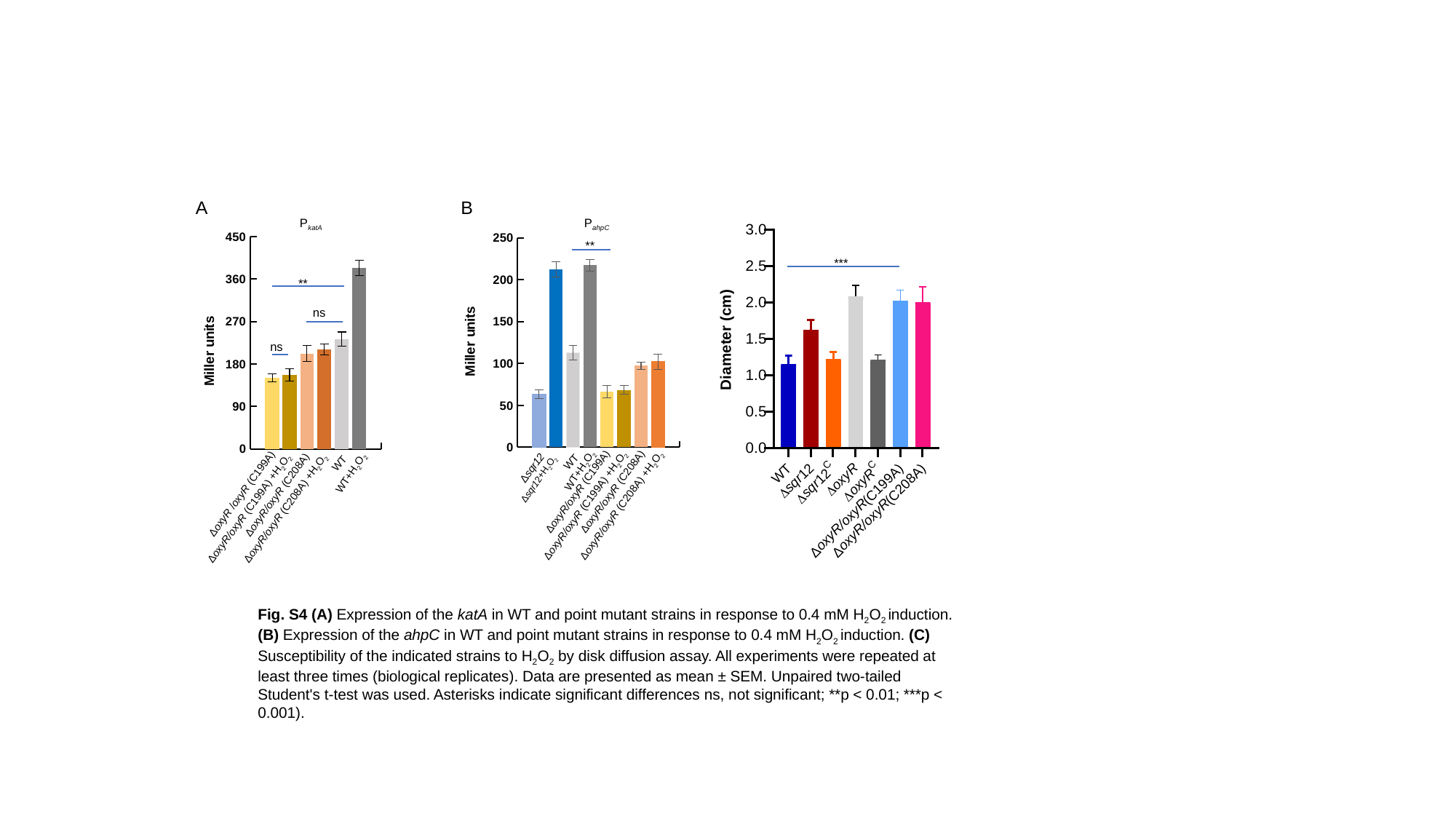

### Chart
| Category | sqr | H | Δsqr | sqrH | WT | WT+H | sqrH | WT |
|---|---|---|---|---|---|---|---|---|A
B
### Chart
| Category | WT | H | WT | Δsqr | sqr | sqrH |
|---|---|---|---|---|---|---| PkatA
 PahpC
**
***
**
ns
ns
Δsqr12
WT
WT
WT+H2O2
Δsqr12+H2O2
WT+H2O2
ΔoxyR/oxyR (C199A)
ΔoxyR/oxyR (C208A)
ΔoxyR /oxyR (C199A)
ΔoxyR/oxyR (C208A)
ΔoxyR/oxyR (C199A) +H2O2
ΔoxyR/oxyR (C208A) +H2O2
ΔoxyR/oxyR (C199A) +H2O2
ΔoxyR/oxyR (C208A) +H2O2
Fig. S4 (A) Expression of the katA in WT and point mutant strains in response to 0.4 mM H2O2 induction.(B) Expression of the ahpC in WT and point mutant strains in response to 0.4 mM H2O2 induction. (C) Susceptibility of the indicated strains to H2O2 by disk diffusion assay. All experiments were repeated at least three times (biological replicates). Data are presented as mean ± SEM. Unpaired two‑tailed Student's t-test was used. Asterisks indicate significant differences ns, not significant; **p < 0.01; ***p < 0.001).

## Slide 5
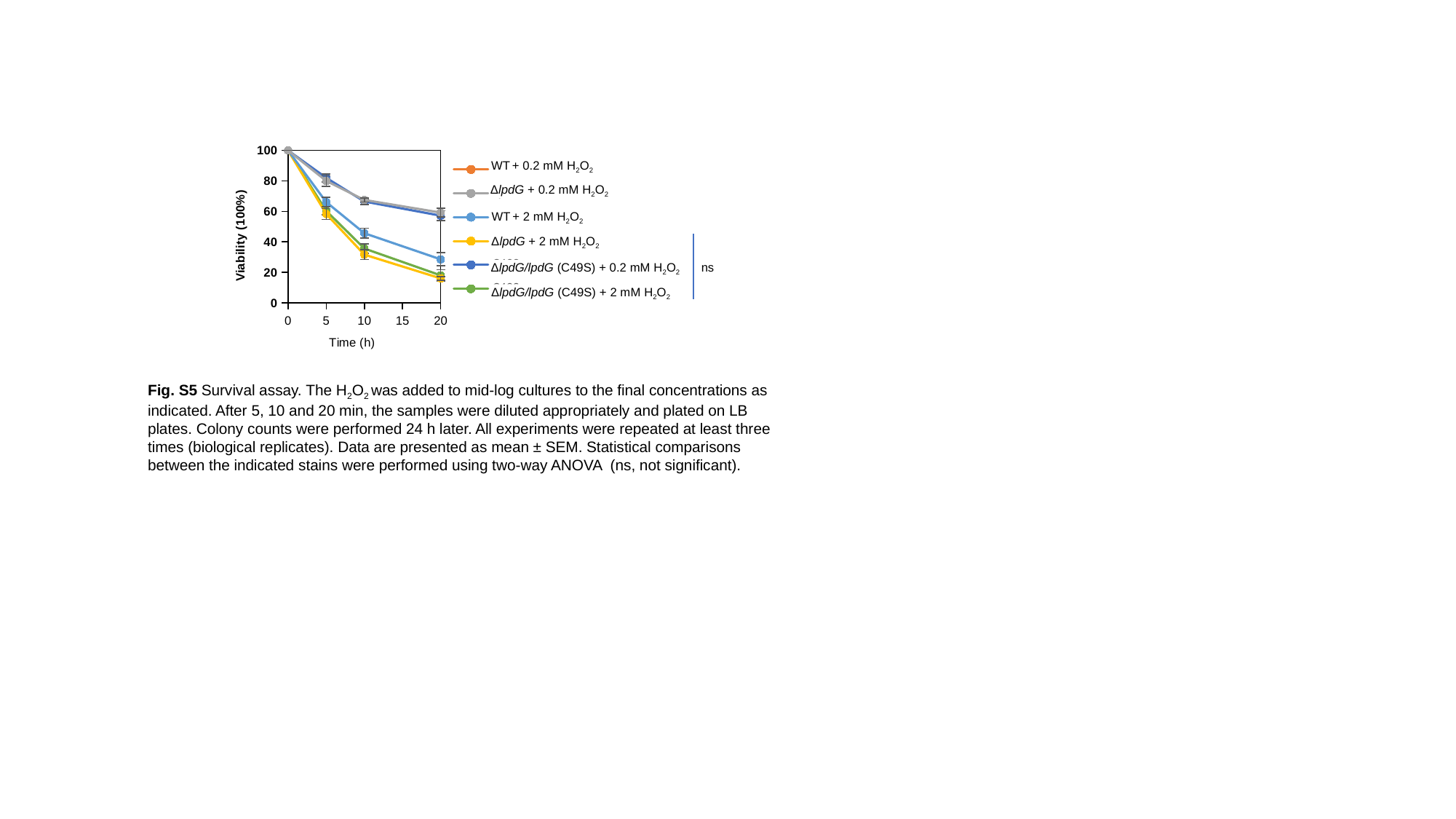

### Chart
| Category | WT-1 | LpdG-1 | WT-2 | LpdG-2 | C49S | C49S |
|---|---|---|---|---|---|---|WT + 0.2 mM H2O2
ΔlpdG + 0.2 mM H2O2
WT + 2 mM H2O2
ΔlpdG + 2 mM H2O2
ns
ΔlpdG/lpdG (C49S) + 0.2 mM H2O2
ΔlpdG/lpdG (C49S) + 2 mM H2O2
Fig. S5 Survival assay. The H2O2 was added to mid-log cultures to the final concentrations as indicated. After 5, 10 and 20 min, the samples were diluted appropriately and plated on LB plates. Colony counts were performed 24 h later. All experiments were repeated at least three times (biological replicates). Data are presented as mean ± SEM. Statistical comparisons between the indicated stains were performed using two‑way ANOVA (ns, not significant).

## Slide 6
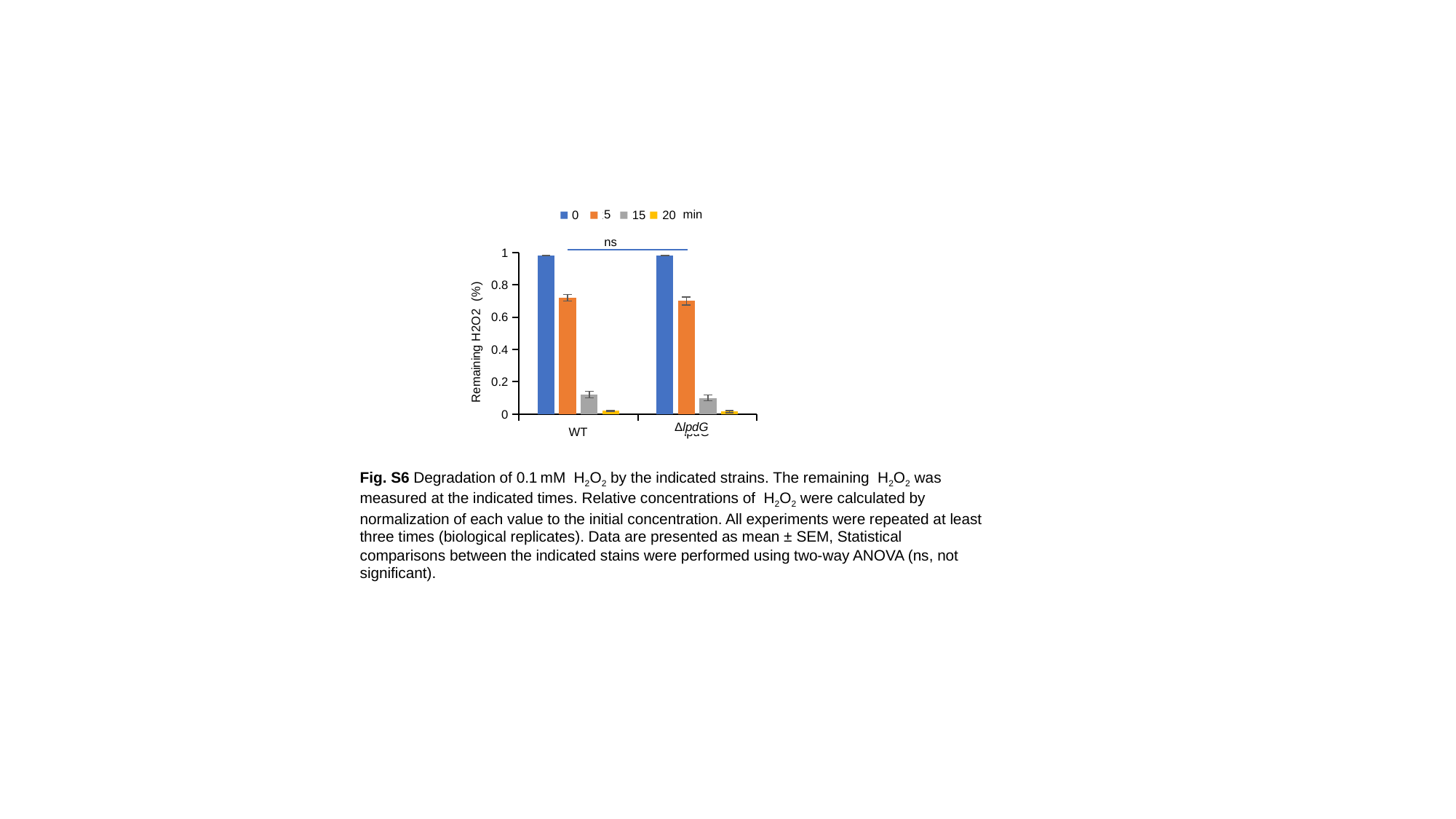

### Chart
| Category | 0 | 1 | 3 | 5 |
|---|---|---|---|---|
| WT | 0.98 | 0.72 | 0.12 | 0.02 |
| lpdG | 0.98 | 0.7 | 0.1 | 0.015 |5
min
20
15
ns
ΔlpdG
Fig. S6 Degradation of 0.1 mM  H2O2 by the indicated strains. The remaining  H2O2 was measured at the indicated times. Relative concentrations of  H2O2 were calculated by normalization of each value to the initial concentration. All experiments were repeated at least three times (biological replicates). Data are presented as mean ± SEM, Statistical comparisons between the indicated stains were performed using two‑way ANOVA (ns, not significant).
