# Supplementary material for: Endogenous Sulfane Sulfur Mediates the Oxidative Stress Response Process in Pseudomonas aeruginosa
Source: Antioxidants (Basel). 2026 May 31;15(6):696. doi: 10.3390/antiox15060696 (PMC13295329; doi:10.3390/antiox15060696)
Supplement: Supplementary file 1 [file antioxidants-15-00696-s001.zip › Table S1 primer.pdf]

Table S1 The primers used in this study.

**Mutagenesis**	
PA2566-5O	CGAATACCTCGTCCGCACC
PA2566-5I	GACGATGACGATTCGTTGCATG
PA2566-3O	.CTCCAGATCCGCACGATCTC
PA2566-3I	CATCGCTGCCGAGATTCGTC
PA2345-5O	GTACTGGAACGCCATGCTCAAG
PA2345-5I	GATGTCGGTGGTGAGTTCTG
PA2345-3O	CGGCGCAATACCAGTTC
PA2345-3I	GTACTGGAACGCCATGCTCAAG
PA5344-5O	GTGGATCCGGTTGGCATACAG
PA5344-5I	CTCGTCCTCGAGCTTCTTC
PA5344-3O	CTTCGGCCAGTTCCTCGATG
PA5344-3I	GGAACAACCGCAAATAGCATG
PA1857-5O	CATCGATCCGAACAGCAT
PA1857-5I	GGATGGCGGCAACGTAAC
PA1857-3O	GAAGATATGGGTGAACTGCTTGAT
PA1857-3I	.GTGAATGGCCATGCCATC
PA4236-5O	TGGAACTGTTGAAAACGCCGAAC
PA4236-5I	CTTCTCGAGGAACCACACGT
PA4236-3O	CTTCCTCAAGCCGTTGCTG
PA4236-3I	CTGTTCCATCTGTTCACCGAC
PA4613-5O	GAAGTCGAGTACGGTCTGAATGAAC
PA4613-5I	TGCACGGACAGCGACAGGAG
PA4613-3O	CATGACGGACCTCCTCTGAC
PA4613-3I	CAAGTACATCATGCTTTCCTACTTCTAC
PA0139-5O	GTACCTGAACGCCAACCAG
PA0139-5I	CACAACGGCAAGTTCATCGAG
PA0139-3O	GTTCATGATGTCGACCTCGTA

PA0139-3I	CTTCGTGATCAACCCGGAAG
**Complementation**	
Sqr1-F	ATGCAACGAATCGTCATCGTC
Sqr1-R	TCAAAGCATTCCACGGGCTAC
Sqr2-F	ATGCAGAGCGCTAACGCTTC
Sqr2-R	CTACTTGAGCATGGCGTTCCAG
LpdG-F	ATGAGCCAGAAATTCGACGTG
LpdG-R	TCAGCGCTTCTTGCGGTTG
OxyR-F	ATGACCCTCACCGAACTG
OxyR-R	TCATGCTATTTGCGGTTGTTC
KatB-F	ATGAACCCTTCCCTGAACG
KatB-R	TCAATCCTGGAGCTTCGC
***lac*Z reporters**	
P_{sqr1}-F	CTCTTCCTGTGGTGGCTG
P_{sqr1}-R	GACTTCTCCTTAACCCTGAG
P_{sqr2}-F	CTCTGGCAGTTCGGATAGGTGG
P_{sqr2}-R	GGGAGTTCCTTCGACGATCC
P_{katA}-F	CTGCCGAATAAGGCATCTG
P_{katA}-R	GCCGTTGAGGAGAGAGTAA
P_{ahpC}-F	CTGTCGATTCCGGCCAAC
P_{ahpC}-R	CAGTCGTTCCTCTCTCAGTTGAATG
P_{katB}-F	CAACTTCGGCAACTTGGAAC
P_{katB}-R	GGAAGAGCTCCTAATGGCTTG
P_{lpdG}-F	GCACCTTCACCATCTCCAAC
P_{lpdG}-R	ATCTCAATCCTTATTCGGGTGAAAC
